# Clinical benefit and risk of elemene in cancer patients undergoing chemotherapy: a systematic review and meta-analysis

**DOI:** 10.3389/fphar.2023.1185987

**Published:** 2023-08-02

**Authors:** Yanhong Pan, Panting Wan, Li Zhang, Cuirong Wang, Yijun Wang

**Affiliations:** ^1^ Department of Pharmacy, The Second Affiliated Hospital of Nanjing Medical University, Nanjing, China; ^2^ Jiangsu Key Laboratory for Pharmacology and Safety Evaluation of Chinese Materia Medica, School of Pharmacy, Nanjing University of Chinese Medicine, Nanjing, China

**Keywords:** elemene, chemotherapy, cancer patients, efficacy, side effects, variables

## Abstract

**Introduction:** Elemene injection and oral emulsion, known as elemene, have been utilized have been used in adjuvant therapy for cancer patients in China for more than 20 years. In order to evaluate the efficacy and potential risks of the treatments in cancer patients undergoing chemotherapy, a system review and meta-analysis were conducted. Additionally, the factors that may influence the outcomes were also explored.

**Methods:** A comprehensive search was conducted across various databases including PubMed, Cochrane Library, Web of Science, EMBASE, CKNI, Wan Fang, and VIP databases. Meta-regression, subgroup, and sensitivity analyses were conducted to explore the heterogeneity. GRADE system and TSA were used to assess the strength of evidence and robustness of the results.

**Results:** The pooled data showed that combination with elemene could improve the response rate (RR:1.48, 95%CI:1.38–1.60, *p* < 0.00001), disease control rate (RR:1.20, 95%CI:1.15–1.25, *p* < 0.00001), the rate of quality-of-life improvement and stability (WMD:1.31, 95% CI:1.12–1.53, *p* = 0.0006), immune function (CD4^+^/CD8^+^: WMD:0.33, 95% CI:0.24–0.42, *p* < 0.00001), survival rate (1-year, RR:1.34, 95% CI:1.15–1.56, *p* = 0.0002; 2-year, RR:1.57, 95% CI:1.14–2.16, *p* = 0.006), and decrease the prevalence of most chemotherapy-induced side effects, especially leukopenia (Ⅲ-Ⅳ) (RR:0.46, 95% CI:0.35–0.61, *p* < 0.00001), thrombocytopenia (RR:0.86, 95% CI:0.78–0.95, *p* = 0.003), and hemoglobin reduction (RR:0.83, 95% CI:0.73–0.95, *p* = 0.007). However, the administration of elemene has been found to significantly increase the incidence of phlebitis in patients undergoing chemotherapy (RR:3.41, 95% CI:1.47–7.93, *p* = 0.004). Meta-regression and subgroup analyses discovered that the outcomes were rarely influenced by CR, CT, and dosage of elemene (DE) but the cycle number of elemene (CNE) and TT were the main sources of heterogeneity.

**Discussion:** As the treatment time and the number of cycles increased, the efficacy of the elemene combination decreased across various aspects. Thus, shorter duration and fewer cycles are recommended.

## Introduction

Cancer is a serious health problem threatening human life all over the world. According to the survey, more than 1.6 million people are diagnosed with cancer and 1.2 million people died of it every year in China (Fan et al., 2014). Even in developed countries, such as the United States, more than 1.8 million new cancer cases and 0.6 million cancer deaths occurred in 2021 (Siegel et al., 2021). Chemotherapy is one of the main treatments for cancer since 1940, which can effectively kill cancer cells. However, no selective killing effect of these drugs caused inevitable body damage during the treatments. Patients frequently experience hair loss, digestive tract reactions, myelosuppression, liver and kidney dysfunction, and other adverse effects. Some patients even die of severe toxic reactions induced by chemotherapy drugs (Diasio and Offer, 2022). Multidrug resistance (MDR) is another problem that limited its application in clinics. Metabolism of xenobiotics, efflux of drugs, growth factors, stress-associated cellular states, and plasticity of cancer cells are involved in MDR (Bukowski et al., 2020; Jewer et al., 2020; Zhang K. et al., 2022). Therefore, the development of new treatments to overcome these disadvantages is quite necessary.

In recent years, active ingredients derived from natural plants have attracted the attention of researchers and developed due to their anticancer activity and the richness of candidate resources. β-elemene, the predominant non-cytotoxic anticancer component of *Curcuma wenyujin* Y.H.Chen & C.Ling and *Curcuma zedoaria* (Christm.) Roscoe (Tao et al., 2016), has been reported to inhibit the proliferation, metastasis, and metabolism of cancer cells, induce apoptosis, and regulate immunity (Pan et al., 2019; Cheng et al., 2022; Kong et al., 2022). It can improve the sensitivity of cancer cells to radiotherapy and chemotherapeutic drugs without myelosuppression and hepatorenal toxicity (Liu et al., 2015; Mu et al., 2016; Zhou et al., 2016; Liu et al., 2020). Elemene oral emulsion (85% β-elemene) and elemene injection (85% β-elemene) collectively referred to as elemene in this study were approved by the China Food and Drug Administration (CFDA) for the therapy of various cancer (Bai et al., 2021). Especially elemene injection has been used in clinical adjuvant therapy for more than 20 years in China. Numerous studies have reported that the incorporation of elemene injection or oral emulsion alongside chemoradiotherapy can mitigate side effects and improve the overall quality of life (Chang et al., 2017; Wang et al., 2019). However, conclusions diverge when it comes to disease control rate (DCR), response rate, and survival rate (Zeng et al., 2011; Zheng et al., 2014; Lei et al., 2018). This discrepancy can be attributed to various factors, including the specific cancer type, sample size (SZ), chemotherapy regimens (CR), treatment time (TT), cycle number of elemene (CNE), and dosage of elemene (DE). Furthermore, few studies have comprehensively evaluated the advantages and potential risks associated with the combined use of elemene and chemotherapy. Therefore, the purpose of this study was to assess the clinical benefit and potential hazards associated with the administration of elemene to cancer patients undergoing chemotherapy in terms of response rate, DCR, side effects, quality of life, survival rate, and immune function, and to look for possible causes.

## Methods

### Protocol and registration

This research was guided by the Preferred reporting items for systematic review and meta-analysis protocols (PRISMA-P) 2015 (Shamseer et al., 2015) and registered at PROSPERO (http://www.crd.york.ac.uk/PROSPERO). The registration number is CRD42022330190.

### Search strategy

Electronic literature in Chinese and English that related to elemene, chemotherapy, and their items were searched in PubMed, Cochrane Library, Web of Science, EMBASE, CKNI, Wan Fang, and VIP databases from inception to April 2022. The literature search was finished by the two independent reviewers C.R.W. and L.Z. The search concepts were shown as follows:

For the English databases: 1. Elemene OR ELE OR Elemene Emulsion OR Elemene Injection AND 2. Chemotherapy OR Chemical therapy. For the Chinese databases: 1. Lanxiangxi (Elemene/ELE) OR Lanxiangxi zhusheye (Elemene Injection) OR Lanxiangxi ru (Elemene Emulsion) AND 2. Hualiao (Chemotherapy/Chemical therapy), and their related terms as MeSH terms, title, and abstract.

### Inclusion and exclusion criteria

Inclusion criteria: 1) Patients were diagnosed with cancer by pathology, cytology, or imaging; 2) Clinical trials; 3) Studies comparing the combination of elemene and chemotherapy with the same chemotherapy; 4) Studies have reported more than one of the following primary or secondary outcomes.

Exclusion criteria: 1) Studies lacking information on cancer patient diagnostics; 2) Nonclinical studies including observational studies, systematic reviews, letters, editorials, clinical guidelines, and commentaries; 3) Studies lacking chemotherapy-only group or combination group; 4) Studies failing to report at least one of the following primary or secondary outcomes.

### Data extraction

Data extraction was carried out by two researchers Y.H.P. and P.T.W. First, the quality of journals was evaluated and the references were screening the title and abstract to remove duplicate and unrelated studies. Then, the studies in accordance with the inclusion criteria were identified by reading the full text. When disagreements arise, the third reviewer Y.J.W. was discussed to reach a consensus. Extracted data included the basic characteristics, such as cancer type, sample size, treatment time, intervention, and outcomes.

### Primary outcome

Response rate, adverse effects, Karnofsky Performance Status (KPS), quality of life improvement and stability rate, and immunocyte.

### Secondary outcome

DCR, survival rate, and lung cancer symptom scale observer scale (LCSS).

### Risk of bias and quality assessment

Cochrane risk assessment tool was used to assess the risk of bias in the included studies by two independent researchers Y.H.P. and P.T.W., and any conflicts were resolved through negotiation. Review Manager 5.3 software was used to record the seven domains: random sequence generation (selection bias), allocation concealment (selection bias), masking of participants and personnel (performance bias), masking of outcome assessment (detection bias), incomplete outcome data (attrition bias), selective reporting (reporting bias), and other bias. Included studies were classified as “low,” “high,” and “unclear” risk of bias, colored green, yellow, and red and presented as “+,” “−,” and “?.” GRADEproflier 3.2.2 software was utilized to evaluate the quality of evidence, and outcomes were rated as “high,” “moderate,” “low,” and “very low” (Guyatt et al., 2011).

### Data synthesis and statistical analysis

Review Manager 5.3 and Stata/MP 14.0 software were used. Continuous outcomes were analyzed using Weighted mean difference (WMD) and 95% confidence interval (CI) and dichotomous outcomes as risk ratios (RR) and 95% CI. *p*-value and I^2^ statistics were used to check the heterogeneity of studies. If I^2^ <50% or *p* > 0.1, a fixed-effects model was applied (Higgins and Thompson, 2002). Otherwise, a random-effects model was used. Publication bias for the same outcome which included more than 10 studies was evaluated by Funnel plots. A sensitivity analysis was performed to evaluate the stability of the results by eliminating the studies one by one (Liu et al., 2022). Meta-regression and subgroup analysis were utilized to evaluate what caused the heterogeneity (Zhu et al., 2022).

### Trial sequential analysis

The TSA software (version 0.9.5.10 Beta) was utilized to evaluate the robustness of the findings in cases where the number of included studies exceeded four. The required information size (RIS) was calculated according to a type I error value of 5%, a power of 80, and a relative risk reduction based on studies with low bias. The reliability of the result was established if the cumulative sample size reached the RIS or the cumulative Z curve intersected the monitoring boundary (Zhang L. et al., 2022).

## Results

### Study selection and characteristic information

**FIGURE 1:**
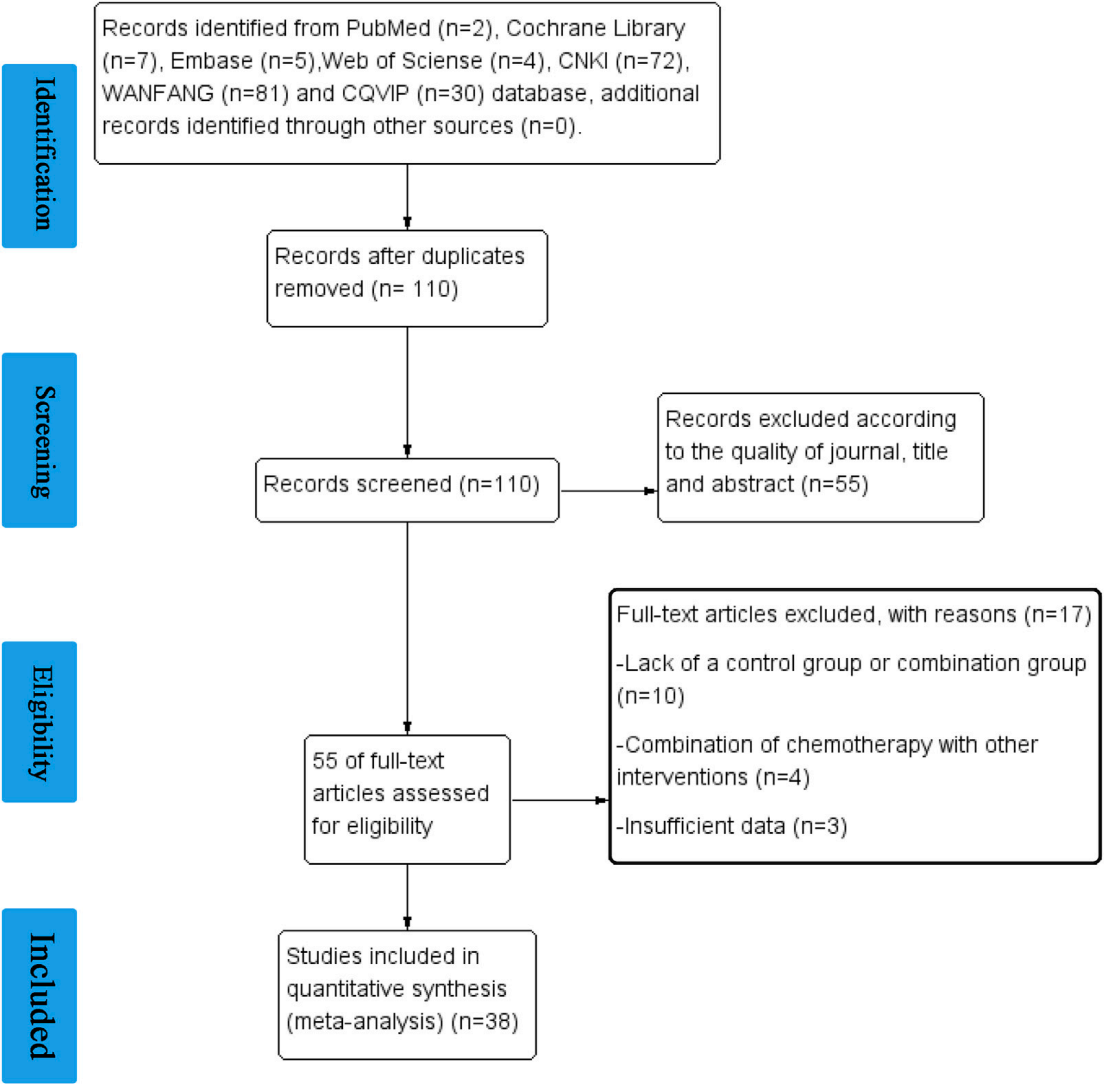
Flow diagram of the study selection.

As shown in Figure 1, we achieved 2, 7, 5, 4, 72, 81, and 30 records from Pubmed, Cochrane Library, EMBASE, Web of Science, CNKI, WANFANG, and VIP databases, respectively. 110 studies were identified after removing duplication. After evaluating the journal’s quality and reading the title and abstract, 55 publications were removed. 17 records were further excluded for the reason of lack of a control group or combination group, a combination of chemotherapy with other interventions, and insufficient data. 38 clinical studies, including 2709 patients, 12 types of cancer, and 35 chemotherapy regimens were finally chosen for this study (Zhang, 1997; Qin et al., 1998; Zhao et al., 1999; Zheng et al., 1999; Liu, 2000; Chen et al., 2005; Chen et al., 2008; Gu et al., 2009; Zhou et al., 2009; Zhou, 2010; Fan et al., 2011; Lu and Zhao, 2011; Zeng et al., 2011; Chen et al., 2012; Wang et al., 2012; Zhao et al., 2012; Zhang et al., 2013a; Feng and Zhao, 2013; Huang et al., 2013; Song et al., 2015; Xu et al., 2017; Lei et al., 2018; Wang et al., 2018; Wu et al., 2018; Xu et al., 2018; Zhao et al., 2018; Shi et al., 2019; Zhang et al., 2019; Zhong and Hao, 2019; Qian et al., 2020; Yu et al., 2020; Fan and Wang, 2021; Han et al., 2021; Liu et al., 2021; Tao et al., 2021). In these studies, elemene, no matter whether administered orally or by injection, was a prescription drug approved for marketing in China. The detailed characteristics and information are summarized in Table 1.

**TABLE 1:** The characteristics of included studies.

Study	Cancer type	Sample size Experimental/Control	Treatment time	Intervention		Outcomes	Dosage of elemene	Cycle number of elemene	Drug delivery of elemene
				Experimental	Control				
Qin et al. (1998)	Lung cancer	27/29	>56 days, <63 days	Elemene + CTV	CTV	1, 2, 3, 4	400 mg/Day	2	Injection
Xu et al. (2018)	Lung cancer	50/50	63 days	Elemene + GP	GP	1, 2, 3, 6, 8	400 mg/Day	3	Injection
Zhou et al. (2009)	Lung cancer	44/40	>63 days	Elemene + Paclitaxel	Paclitaxel	1, 2, 3	400 mg/m^2^/Day	4	Injection
Zhang (1997)	Lung cancer	16/17	28 days	Elemene + Cisplatin + VP-16	Cisplatin + VP-16	1	400 mg/m^2^/Day	2	Injection
Song et al. (2015)	Lung cancer	60/60	>63 days	Elemene + NP	NP	1, 3, 5	400 mg/Day	4	Injection
Lei et al. (2018)	Lung cancer	29/29	>42 days	Elemene + TP/GP/PC	TP/GP/PC	1, 2, 4, 7	500 mg/Day	>6	Injection
Zhao et al. (2018)	Lung cancer	35/35	>63 days	Elemene + TP	TP	1, 2, 3, 6, 7	600 mg/Day	4	Injection
Chen et al. (2008)	Lung cancer	68/71	42 days	Elemene + DC	DC	1, 2, 3, 5, 6	400 mg/Day	2–3	Injection
Liu (2000)	Lung cancer	23/20	56–84 days	Elemene + MVP	MVP	1, 3, 6	500 mg/Day	2	Injection
Wang et al (2012)	Lung cancer	31/30	28 days	Elemene + TC	TC	1, 2, 3, 5	500 mg/Day	2	Injection
Zhou (2010)	Lung cancer	36/21	>63 days	Elemene + TP	TP	1, 2, 3, 7	400 mg/m^2^/Day	ND	Injection
Fan and Wang (2021)	Lung cancer	36/36	63 days	Elemene + Pemetrexed + Cisplatin + Gefitinib	Pemetrexed + Cisplatin + Gefitinib	1, 2, 5, 6, 7, 8	400 mg/Day	3	Injection
Chen et al. (2005)	Lung cancer	33/30	56 days	Elemene + Docetaxel	Docetaxel	1, 2, 3, 6	800 mg/Day	2	Injection
Zhang et al. (2019)	Lung cancer	40/33	42 days	Elemene + GP	GP	1, 2, 3, 6	400 mg/Day	3	Injection
Fan et al. (2011)	Gastric cancer	41/40	42 days	Elemene + XELOX	XELOX	1, 2, 3, 7	100 mg/Day	2	Injection
Zeng et al. (2011)	Gastric cancer	25/24	56 days	Elemene + FOLFOX4	FOLFOX4	1, 2, 3, 5, 6	500 mg/Day	4	Injection
Qian et al. (2020)	Gastric cancer	35/36	>42 days	Elemene + SOX	SOX	1, 3, 6	400 mg/Day	2	Injection
Tao et al. (2021)	Gastric cancer	38/38	42 days	Elemene + SOX	SOX	1, 2, 3	176 mg × 3/Day	2	Orally
Yu et al. (2020)	Gastric cancer	45/45	42 days	Elemene + FOLFOX4	FOLFOX4	1, 2, 3	500 mg/Day	3	Injection
Zhong and Hao (2019)	Gastric cancer	30/30	42 days	Elemene + XELOX + Trastuzumab	XELOX + Trastuzumab	1, 2, 7	500 mg/Day	2	Injection
Wang et al. (2018)	Gastric cancer	30/30	>63 days	Elemene + XELOX	XELOX	1, 2, 4, 6	600 mg/Day	6	Injection
Bi et al. (2012)	Gastric cancer	25/24	56 days	Elemene + FOLFOX4	FOLFOX4	1, 2, 3, 5, 6	500 mg/Day	4	Injection
Chen et al. (2022)	Gastric cancer	17/22	63 days	Elemene + Lobaplatin + Capecitabine + Oxaliplatin	Lobaplatin + Capecitabine + Oxaliplatin	3, 6	600 mg/Day	2	Injection
Han et al. (2021)	Breast cancer	56/49	>63 days	Elemene + TAC	TAC	1, 2, 3, 6, 7	400–600 mg/Day	6	Injection
Xu et al. (2017)	Breast Cancer	42/42	>63 days	Elemene + TAC	TAC	1, 3, 7	400–600 mg/Day	6	Injection
Zhao et al. (2012)	Liver cancer	21/20	56 days	Elemene + Cisplatin + 5-Fu + Epirubicin + Mitomycin + Lipiodol	Cisplatin + 5-Fu + Epirubicin + Mitomycin + Lipiodol	1, 2, 3, 5	200 mg/Day	2	Injection
Lu and Zhao (2011)	Liver cancer	31/30	>56 days, <63 days	Elemene + 5-Fu/FUDR + Oxaliplatin + Lipiodol	5-Fu/FUDR + Oxaliplatin + Lipiodol	1, 2, 3, 4, 7	800 mg/Day	2	Injection
Zhao et al. (1999)	Acute myelocytic leukemia	18/12	14 days	Elemene + Ara-C + VP-16	Ara-C + VP-16	1	800 mg/Day	2	Injection
Zheng et al. (1999)	Acute myelocytic leukemia	20/23	12–15 days	Elemene + HA	HA	1, 3	800 mg/Day	2	Injection
Zheng et al. (2014)	Acute myelocytic leukemia	120/121	36–42 days	Elemene + HAA	HAA	1, 3	800 mg/Day	2	Injection
Gu et al. (2009)	Lung cancer, Esophagus cancer, Gastric cancer, Colorectal cancer, Non-Hodgkin’s lymphoma	70/60	42 days	Lung cancer, Elemene + EP/NP; Esophagus cancer, Elemene + CF/DF; Gastric cancer, Elemene + CF/DF/ECF; Colorectal cancer, Elemene + Oxaliplatin + CF/5-Fu; Non-Hodgkin’s lymphoma, Elemene + CHOP	Lung cancer, EP/NP; Esophagus cancer, CF/DF; Gastric cancer, CF/DF/ECF; Colorectal cancer, Oxaliplatin + CF/5-Fu; Non-Hodgkin’s lymphoma, CHOP	1, 2, 3	800 mg/Day	2	Injection
Huang et al. (2013)	Gastric cancer, Colorectal cancer, Liver cancer, Breast cancer, Pancreatic cancer, Ovarian cancer	28/34	14 days	Gastric cancer, Elemene + DCF; Colorectal cancer, Elemene + OLF/FOLFOX4; Liver cancer, Elemene + Sorafenib; Breast cancer, Elemene + CMF; Pancreatic cancer, Elemene + GP; Ovarian cancer, Elemene + TC	Gastric cancer, DCF; Colorectal cancer, OLF/FOLFOX4; Liver cancer, Sorafenib; Breast cancer, CMF; Pancreatic cancer, GP; Ovarian cancer, TC	1, 3, 5	800 mg/Day	2	Injection
Zhang et al. (2013a)	Multiple Myeloma	13/12	>63 days	Elemene + VAD	VAD	1, 2, 3, 5, 7	400 mg/Day	4	Injection
Feng and Zhao (2013)	Non-Hodgkin lymphoma	38/34	>63 days	Elemene + CHOPE	CHOPE	1, 2, 3	300 mg/Day	ND	Injection
Shi et al. (2019)	Esophageal cancer	21/22	42 days	Elemene + Docetaxel + Oxaliplatin	Docetaxel + Oxaliplatin	1, 2, 3, 4, 5, 7	800 mg/Day	2	Orally
Chen et al. (2012)	Esophageal cancer	18/18	>63 days	Elemene + Paclitaxel	Paclitaxel	1, 2, 3, 5	800 mg/Day	2	Injection
Wu et al. (2018)	Malignant pleural mesothelioma	31/31	≥42 days	Elemene + PC	PC	1, 2, 3, 6	200 mg/m^2^/Day	ND	Injection
Liu et al. (2021)	Colorectal cancer	35/35	>63 days	Elemene + XELOX	XELOX	3, 7	400 mg/Day	6–8	Injection

Outcomes: 1, Response rate, 2, DCR, 3, Adverse effects, 4; KPS, 5, Quality of life improvement and stability rate, 6, Survival rate, 7, Immunocyte, 8, LCSS; TAC, Docetaxel + Cyclophosphamide + Doxorubicin; CTV, Cyclophosphamide + Adriamycin pyranodoxorubicin + Vncristine; GP, Gemcitabine + Cisplatin; CHOPE, Cyclophosphamide + Idarubicin + Vindesine + Dexamethasone + VP-16; XELOX, Oxaliplatin + Capecitabine; NP, Vinorelbine + Cisplatin; VAD, Vincristine + Adriamycin + Dexamethasone; DCF, Docetaxel + Cisplatin + 5-Fu; CMF, Cyclophosphamide + Methotrexate + 5-Fu; TC, Paclitaxel + Carboplatin; FOLFOX6, Oxaliplatin 80–100 mg/m^2^ + Calcium folinate 200 mg/m^2^ + 5-Fu 400 mg/m^2^; PC, Pemetrexed + Cisplatin; TP, Paclitaxel + Cisplatin; DC, Cisplatin + Docetaxel; HA, Harringtonine + Cytarabine; MVP, Mitomycin + Vindesine + Cisplatin; EP, VP-16 + Cisplatin; CF, Carboplatin + 5-Fu; DF, Cisplatin + 5-Fu; ECF, Epirubicin + Cisplatin + 5-Fu; CHOP, Cyclophosphamide + Doxorubicin + Vincristine + Prednisone; OLF, Oxaliplatin 130 mg/m^2^ + Calcium folinate 200 mg/m^2^ + 5-Fu 400 mg/m^2^; FOLFOX4, Oxaliplatin 85 mg/m^2^ + Calcium folinate 200 mg/m^2^ + 5-Fu 400 mg/m^2^; SOX, Oxaliplatin + Tegafur; HAA, Harringtonine + Aclacinomycin + Cytarabine.

### Risk of bias assessment

**FIGURE 2:**
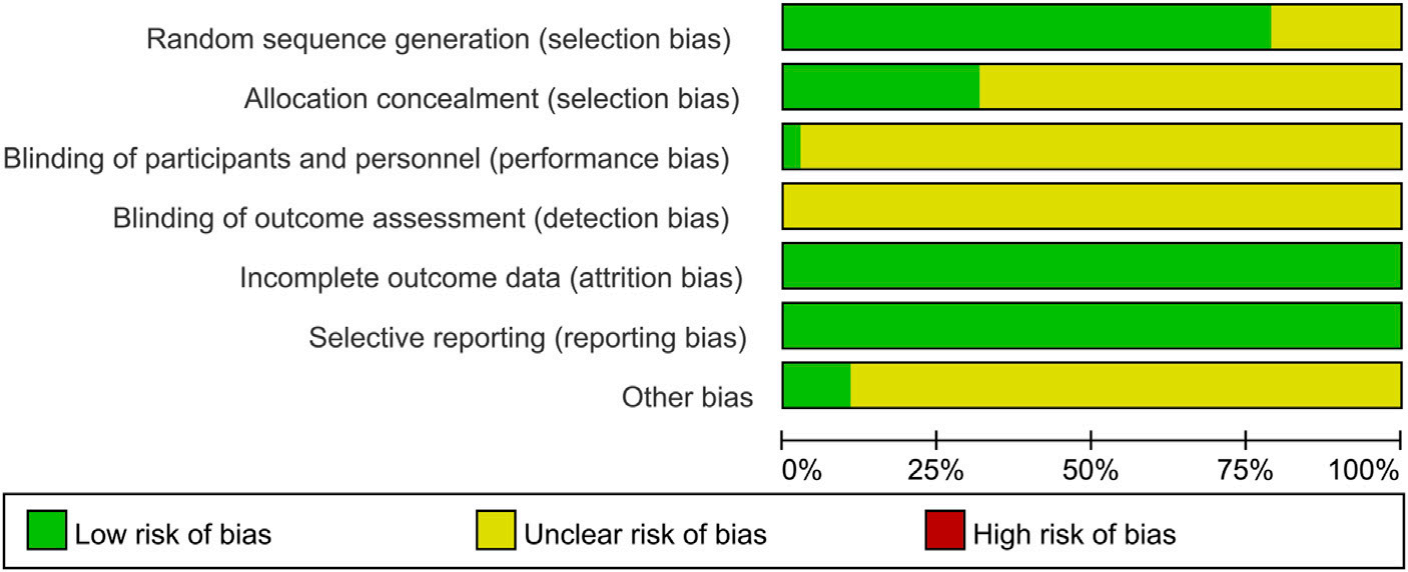
Risk of bias graph.

**FIGURE 3:**
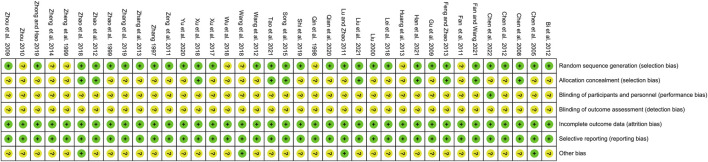
Risk of bias summary.

The risk of bias was assessed and presented in Figure 2 and Figure 3. 78.9% of the studies were randomly designed and 31.6% had low risks of allocation concealment. For attrition bias, all trials were ranked as low risk. However, the majority of the studies did not mention whether the process was double-blind. For reporting bias, all of the studies were ranked as low risk, and for most of them, the presence of other biases was not clearly indicated.

### Elemene improved the response rate and disease control rate of cancer patients treated with chemotherapy

**FIGURE 4:**
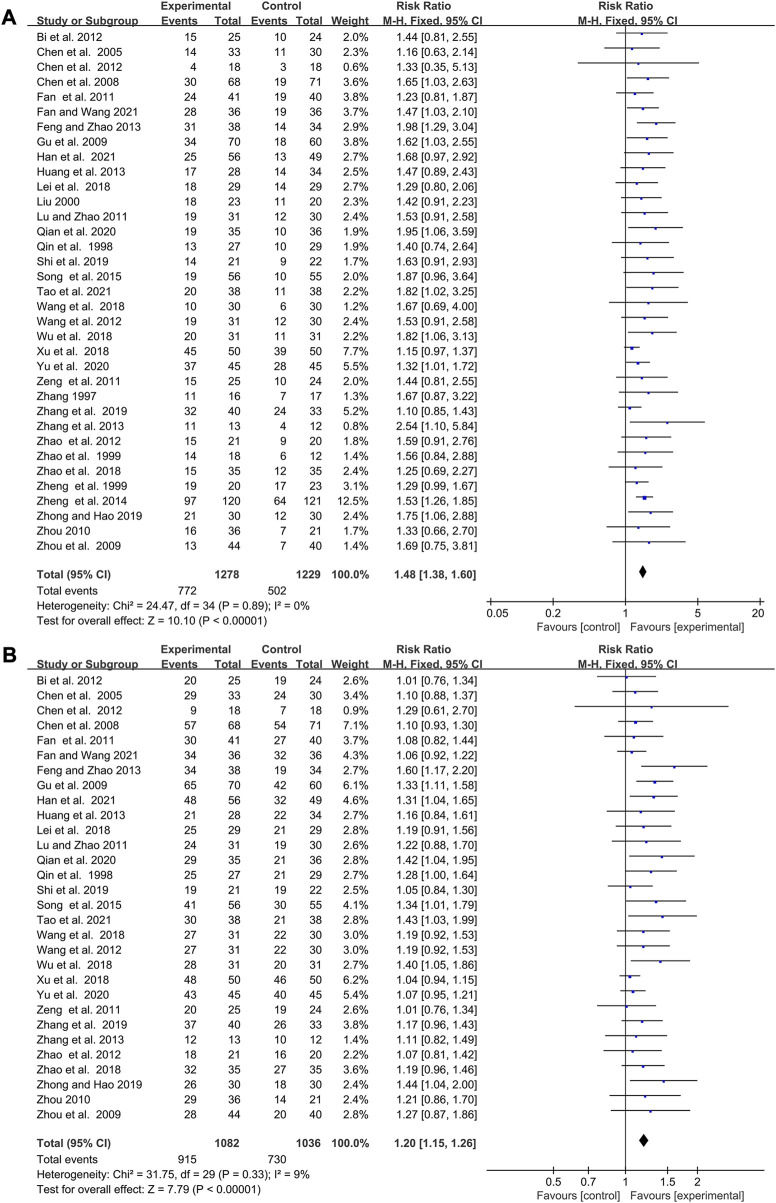
Forest plot displaying the efficacy of elemene on the response rate **(A)** and DCR **(B)** of cancer patients treated with chemotherapy.

35 studies reported changes in response rate while 30 pieces of research revealed variations of DCR after therapy. The studies involving response rate and DCR were homogenous (I^2^ = 0%, *p* = 0.89; I^2^ = 9%, *p* = 0.33 Figures 4A,B), so fixed-effects models were selected for their analysis. The pooled data showed that combining with elemene had a better response rate and DCR than chemotherapy alone (RR:1.48, 95%CI:1.38–1.60, *p* < 0.00001; RR:1.20, 95%CI:1.15–1.25, *p* < 0.00001, Figures 4A,B). The meta-regression analysis showed CNE could moderate the response rate and DCR (*p* = 0.082 and *p* = 0.019, Supplementary Table S1), while SZ, CR, CT, TT, DE, and DDE did not have a significant impact. Subgroup analysis discovered that the improvement of elemene on response rate and DCR might disappear when its cycle number was more than 6 (Supplementary Figures S1A, B). According to the funnel plots for the included studies, we believed that the publication bias was extremely low (Supplementary Figures S3A, B). The sensitivity analysis demonstrated that the combined estimates remained unaffected by any individual study (Supplementary Figures S2A, B). The TSA analysis showed the sample size reached RIS, with the Z curve crossing the conventional and TSA boundaries (Supplementary Figures S6A, B), indicating the robustness of these findings.

### The influence of elemene on the side effects of chemotherapy

**FIGURE 5:**
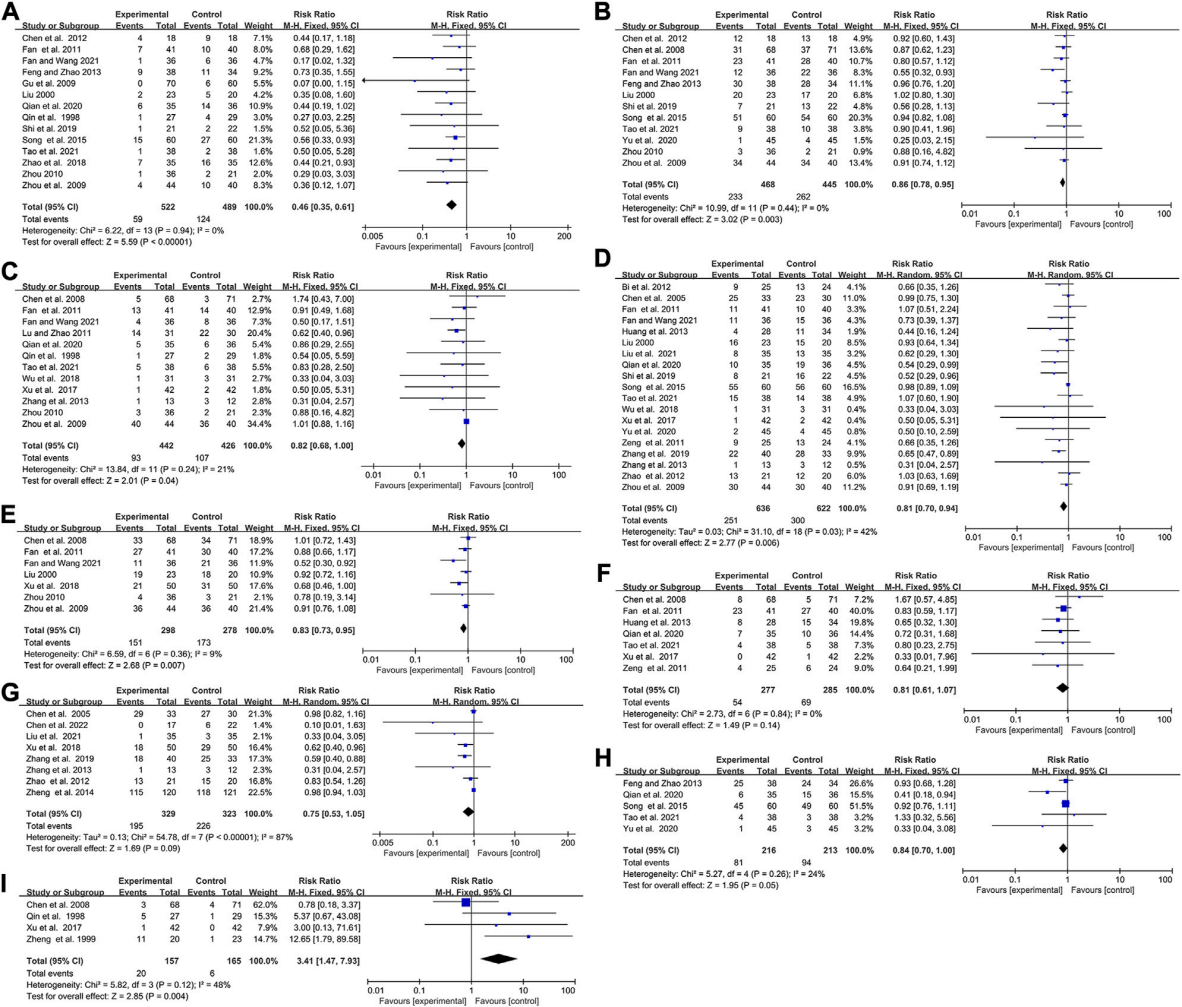
Forest plots showing the effect of combing with elemene on side effects compared with chemotherapy alone **(A)** Leukopenia (Ⅲ-Ⅳ), **(B)** Thrombocytopenia, **(C)**Liver function damage, **(D)** Digestive tract reactions, **(E)** Hemoglobin reduction, **(F)** Neurotoxicity, **(G)** Myelosuppression, **(H)**Anemia, **(I)** Phlebitis.

35 publications with 2397 patients studied the influence of elemene on the adverse reactions of chemotherapy, including leukopenia, thrombocytopenia, and digestive tract reactions (Table 1; Figure 5; Supplementary Figures S4, S5).

There was no heterogeneity in studies involving leukopenia (Ⅲ-Ⅳ), thrombocytopenia, and liver function damage (I^2^ = 0, *p* = 0.94; I^2^ = 0%, *p* = 0.44; I^2^ = 21%, *p* = 0.24). The overall results showed that elemene reduced the incidence of chemotherapy-induced leukopenia (Ⅲ-Ⅳ), thrombocytopenia, and liver function damage in cancer patients (RR:0.46, 95% CI:0.35–0.61, *p* < 0.00001; RR:0.86, 95% CI:0.78–0.95, *p* = 0.003; RR:0.82, 95% CI:0.68–1.00, *p* = 0.04, Figures 5A–C). A random-effects model was applied because of the heterogeneity (I^2^ = 43%, *p* = 0.03, Figure 5D), and an improvement of digestive tract reactions was seen in cancer patients who received elemene in combination with chemotherapy (RR:0.81, 95% CI:0.70–0.94, *p* = 0.006, Figure 5D). However, evidence of publication bias was observed through the presence of asymmetry in the funnel plots shown in Supplementary Figures S3C–F. Meta-regression analysis only discovered a significant association between TT with the prevalence of digestive tract reactions (*p* = 0.043, Supplementary Table S1). Subgroup analysis showed that the prevalence of digestive tract reactions was remarkably reduced by elemene when TT was no more than 42 days (RR:0.70, 95% CI:0.55–0.91, *p* = 0.007, Supplementary Figure S4B), while a slight reduction of the incidence of liver function damage occurred when the CNE value ranged from 2–3 (RR:0.76, 95% CI:0.55–1.03, *p* = 0.08, Supplementary Figure S4A). Sensitivity analysis showed the results were stable (Supplementary Figures S2C–F).

Hemoglobin reduction, neurotoxicity, myelosuppression, anemia, and kidney function damage are also common during chemotherapy. In this research, we found the included clinical studies on hemoglobin reduction, neurotoxicity, and anemia were homogeneous (I^2^ = 9%, *p* = 0.36; I^2^ = 0%, *p* = 0.84, I^2^ = 24%, *p* = 0.26, Figures 5E,F,H), while the ones on myelosuppression and kidney function damage were heterogeneous (I^2^ = 87%, *p* < 0.00001, Figure 5G; I^2^ = 73%, *p* = 0.01; Supplementary Figure S5). The summarized results discovered that the inclusion of elemene was less likely to cause hemoglobin reduction and anemia than chemotherapy alone (RR:0.83, 95% CI:0.73–0.95, *p* = 0.007, Figure 5E; RR:0.84, 95% CI:0.70–1.00, *p* = 0.05; Figure 5H). However, no significant difference was observed in terms of neurotoxicity, myelosuppression and kidney function damage (RR:0.81, 95% CI:0.61–1.07, *p* = 0.14, Figure 5F; RR:0.75, 95% CI:0.53–1.05, *p* = 0.09; Figure 5G; RR:0.59, 95% CI:0.26–1.37, *p* = 0.22; Supplementary Figure S5). These factors were confirmed to be stable through sensitivity analysis (Supplementary Figures S2G–K). Meta-regression analysis also discovered no significant association between hemoglobin reduction, neurotoxicity, anemia, and kidney function damage with variables shown in Supplementary Table S1 (*p* > 0.1), but CNE was the source of heterogeneity of myelosuppression (*p* = 0.018). Subgroup analysis confirmed the combination group exhibited a significantly reduced incidence of myelosuppression and kidney function damage when elemene was administered for only three cycles (RR:0.61, 95% CI:0.45–0.81, *p* = 0.0008, Supplementary Figure S4C; RR:0.42, 95% CI:0.22–0.80, *p* = 0.009; Supplementary Figure S4E), while slightly decreased the occurrence of anemia in patients with gastric cancer (RR:0.53, 95% CI:0.28–1.03, *p* = 0.06, Supplementary Figure S4D). Long-term use of chemotherapy drugs can easily lead to phlebitis, which is also the main adverse reaction of elemene injection, with an incidence of about 10% (Zhai et al., 2018). Unsurprisingly, the pooled results showed that elemene aggravated the incidence of phlebitis in patients undergoing chemotherapy (RR:3.41, 95% CI:1.47–7.93, *p* = 0.004, Figure 5I). Sensitivity analysis found that Chen et al. 2008 influenced this result (Supplementary Figure S3L), which might be related to the use of dexamethasone before chemotherapy in this study.

For leukopenia (Ⅲ-Ⅳ), its Z curve met the RIS and TSA boundary implying the benefit of the combination was conclusive (Supplementary Figure S6C). Although the cumulative Z curves for thrombocytopenia, digestive tract reactions, hemoglobin reduction, and myelosuppression did not reach the RIS, the crossing conventional boundary and TSA boundary suggested that their pooled results were not randomized (Supplementary Figures S6D, F, G, I). However, TSA for liver function damage, neurotoxicity, and anemia showed the cumulative Z value missed the RIS (6218, 4897, 3914, respectively) and TSA boundary, which suggested the conclusion needed to be confirmed by subsequent studies (Supplementary Figures S6E, H, J).

Based on the above results, we believed that elemene could reduce the occurrence of most chemotherapy-induced side effects, especially leukopenia, thrombocytopenia, and digestive tract reactions. However, the cycle number of elemene must be controlled.

### The efficacy of elemene combined with chemotherapy on the percentage of immunocytes

**FIGURE 6:**
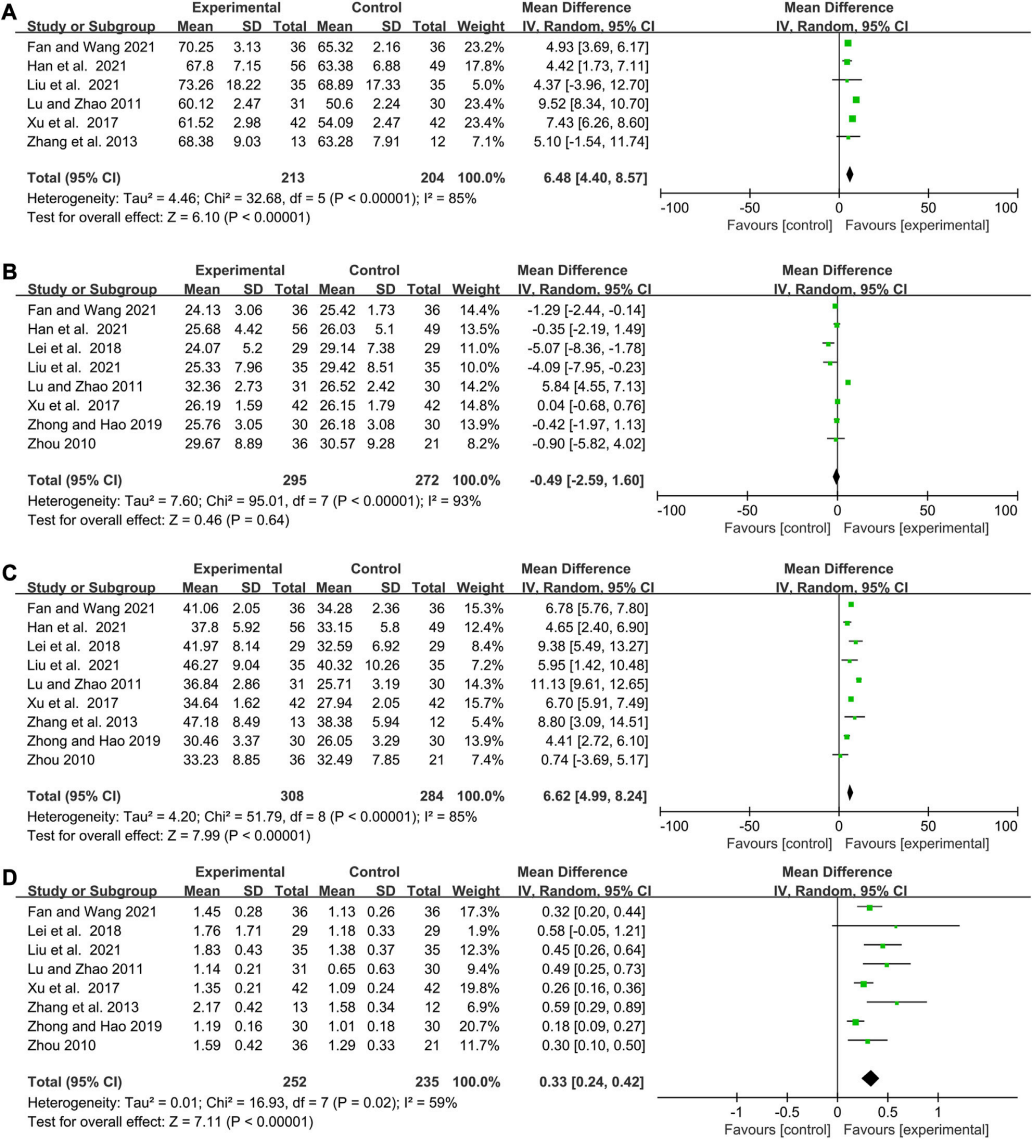
Forest plots showing the efficacy of elemene combined with chemotherapy on the percentage of **(A)** CD3^+^ T cells, **(B)** CD8^+^ T cells, **(C)** CD4^+^ T cells, and **(D)** CD4^+^/CD8^+^ T cells.

Studies about CD3^+^ T cells, CD8^+^ T cells, CD4^+^ T cells, and CD4^+^/CD8^+^ ratio were statistically heterogeneous (I^2^ = 85%, *p* < 0.00001; I^2^ = 93%, *p* < 0.00001; I^2^ = 85%, *p* < 0.00001; I^2^ = 59%, *p* = 0.02, Figure 6). Combining with elemene increased the percentage of CD3^+^ T cells, CD4^+^ T cells and CD4^+^/CD8^+^ ratio of chemotherapy patients (WMD:6.48, 95% CI:4.40–8.57, *p* < 0.00001, Figure 6A; WMD:6.62, 95% CI:4.99–8.24, *p* < 0.00001; Figure 6C; WMD:0.33, 95% CI:0.24–0.42, *p* < 0.00001; Figure 6D). However, it had no impact on the proportion of CD8^+^ T cells (WMD: 0.49, 95% CI: 2.59–1.60, *p* = 0.64, Figure 6B). The funnel plot also suggested a publication bias for studies about CD4^+^ T cells (Supplementary Figure S3G). Sensitivity and meta-regression analysis did not identify any studies or variables that could influence the results (Supplementary Figures S3M–P, *p* > 0.1; Supplemenytary Table S1). Subgroup analysis showed that the presence of CNE CT, DE, and TT contributed to the heterogeneity observed in the studies regarding the proportion of CD3^+^ T cells, CD8^+^ T cells, CD4^+^ T cells, and the CD4^+^ T cells to CD8^+^ T cells ratio, respectively (Supplementary Figures S4F–I). Elemene could not significantly enhance the percentage of CD3^+^ T cells in chemotherapy patients after 6 cycles (WMD:4.37, 95% CI: 3.96–12.70, *p* = 0.30 Supplementary Figure S4F). However, it did increase the percentage of CD8^+^ T cells in liver cancer patients, while decreasing their percentage in colorectal cancer patients (WMD:5.84, 95% CI:4.55–7.13, *p* < 0.00001; WMD: 4.09, 95% CI: 7.95 to −0.23, *p* = 0.04, Supplementary Figure S4G). Furthermore, subgroup analysis demonstrated that elemene remarkably elevated the CD4^+^ T cells to CD8^+^ T cells ratio in chemotherapy patients when the treatment time exceeded 42 days (WMD:0.36, 95% CI:0.27–0.45, *p* < 0.00001, Supplementary Figure S4I), suggesting that elemene had the potential to improve the immune function of chemotherapy patients. The cumulative Z curves obtained from TSA indicated that the results were robust, as they reached the RIS or TSA boundaries, except for CD8^+^ T cells which lost the RIS, conventional boundary, and TSA boundary (Supplementary Figures S6K–N). However, it is important to note that the result regarding CD8^+^ T cells may change in the future with a larger sample size.

### The impact of elemene on the quality of life among cancer patients undergoing chemotherapy

**FIGURE 7:**
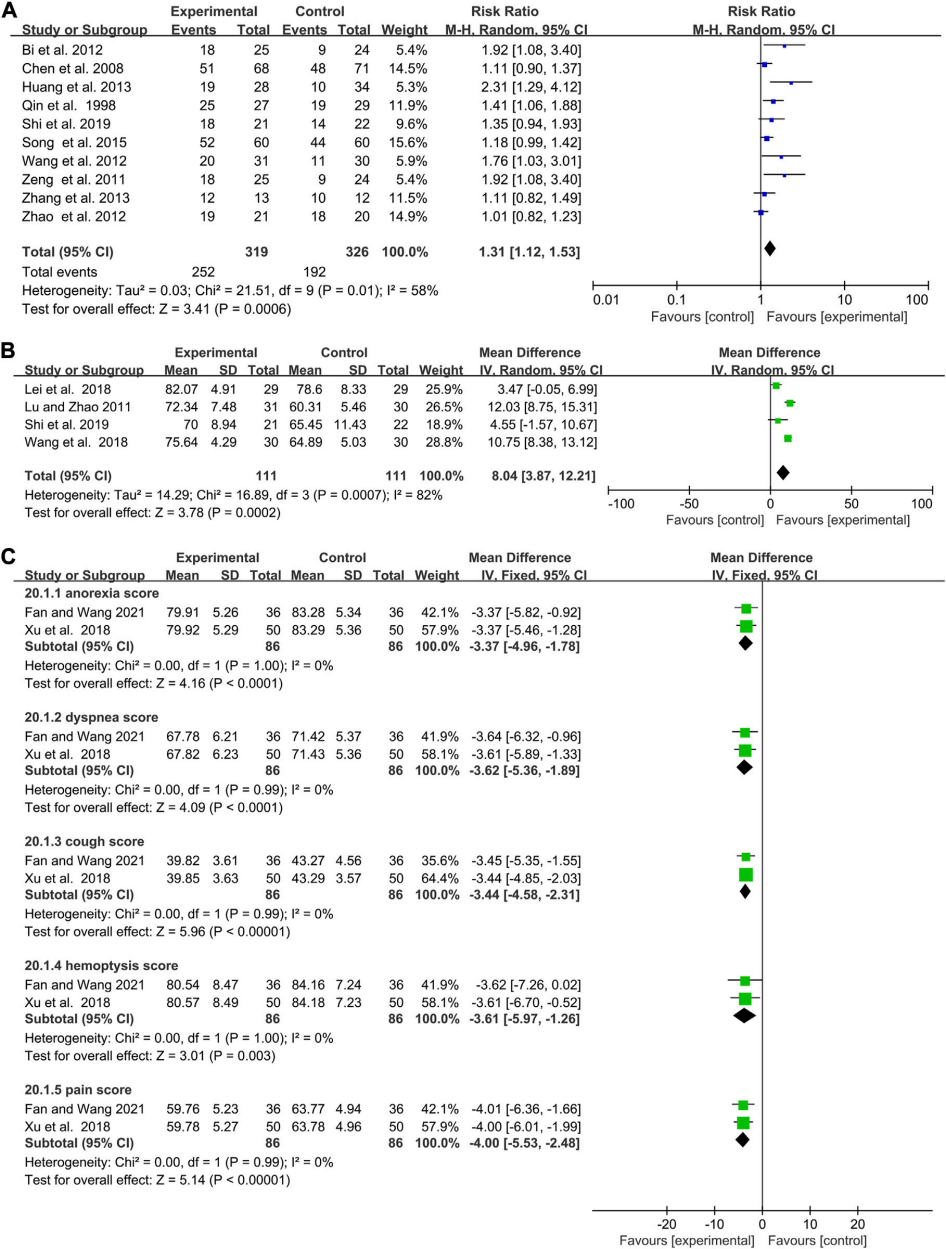
Forest plots of studies evaluating the quality of life of cancer patients **(A)** Quality of life improvement and stability rate, **(B)** KPS **(C)** LCSS.

The rate of improvement and stability in quality of life and KPS are commonly used to evaluate the quality of life of cancer patients (Vaitkiene et al., 2019). A higher score on these measures indicates better overall health status and greater tolerance for the side effects of treatment. The pooled data showed that elemene was able to elevate the rate of improvement and stability in quality of life, as well as KPS, among chemotherapy patients (WMD:1.31, 95% CI:1.12–1.53, *p* = 0.0006, Figure 7A; WMD:8.04, 95% CI:3.87–12.21, *p* = 0.0002; Figure 7B). Sensitivity analysis and TSA analysis showed that the results were stable and conclusive (Supplementary Figures S2Q–R; Supplementary Figure S6O), despite the heterogeneity observed in the included clinical trials (I^2^ = 58%, *p* = 0.01, Figure 7A; I^2^ = 82%, *p* = 0.0007; Figure 7B). Meta-regression analysis discovered CR was associated with quality-of-life improvement and stability rate (*p* = 0.085, Supplementary Table S1). Subgroup analysis revealed that elemene was more likely to increase the rate of improvement and stability in quality of life among cancer patients treated with cisplatin and docetaxel/vinorelbine, FOLFOX4, CTV, or paclitaxel and carboplatin (WMD:1.15, 95% CI:1.00–1.32, *p* = 0.05; WMD:1.19, 95% CI:1.28–2.88, *p* = 0.002; WMD:1.41, 95% CI:1.06–1.88, *p* = 0.02; WMD:1.76, 95% CI:1.03–3.01, *p* = 0.04, Supplementary Figure S4J). Its publication bias was shown in Supplementary Figure S4H. Additionally, subgroup analysis of KPS based on CNE indicated that the combination of elemene for a maximum of 6 cycles was more effective in enhancing KPS (WMD:10.09, 95% CI:6.99–13.20, *p* < 0.00001, Supplementary Figure S4K). LCSS was often used to evaluate the quality of life of lung cancer. As expected, elemene could lower the scores of anorexia, cough, dyspnea, hemoptysis, and pain in lung cancer patients receiving chemotherapy (*p* < 0.0001), and these studies of these outcomes were homogeneity (I^2^ = 0%, *p* = 1.00; I^2^ = 0%, *p* = 0.99; I^2^ = 0%, *p* = 0.99; I^2^ = 0%, *p* = 1.00; I^2^ = 0%, *p* = 0.99; Figure 7C). Therefore, combining with elemene could improve the quality of life among chemotherapy patients.

### The efficacy of elemene on the survival rate of lung cancer patients treated with chemotherapy

**FIGURE 8:**
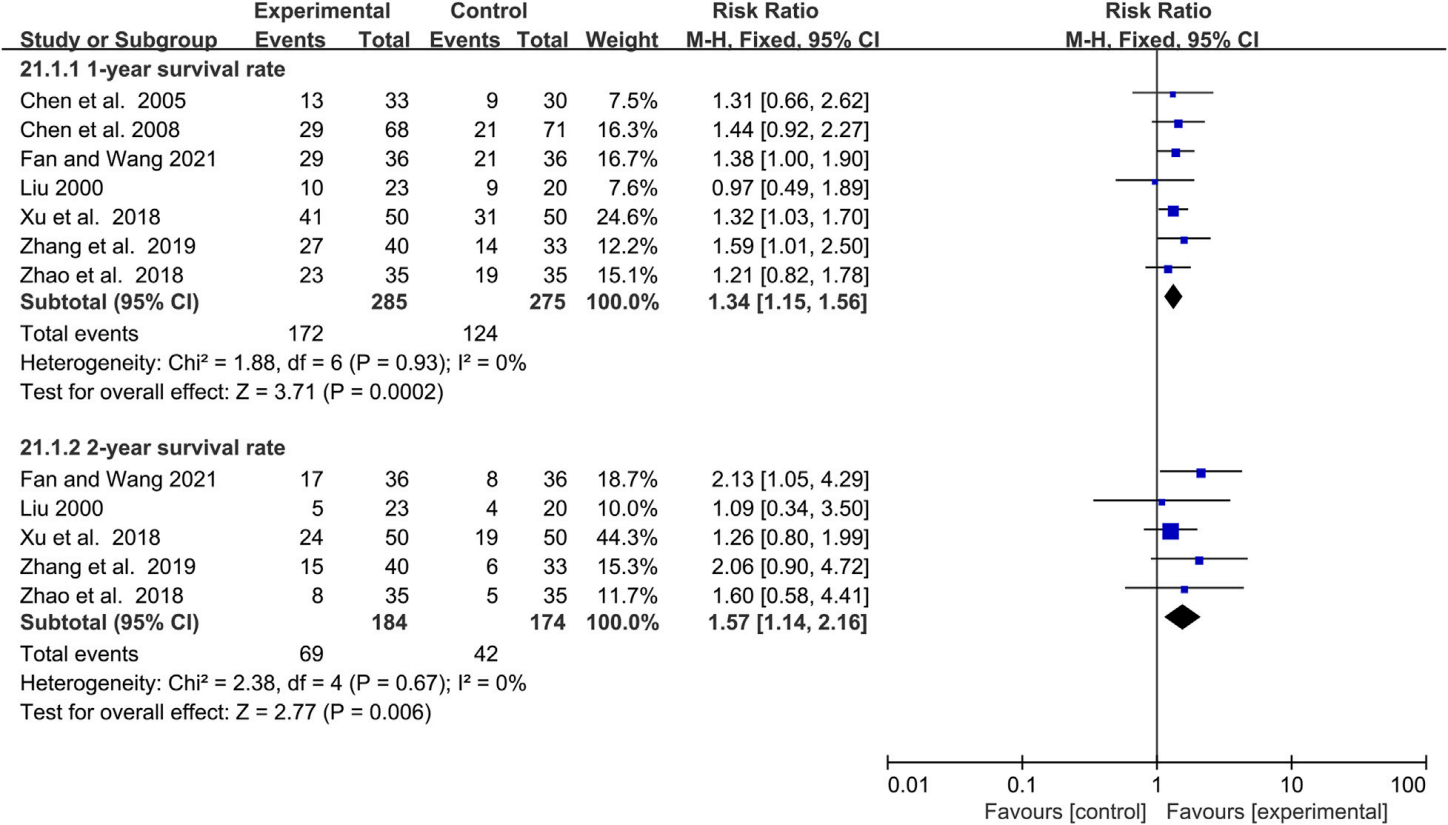
The efficacy of elemene on 1-year survival rate and 2-year survival rate of lung cancer patients undergoing chemotherapy.

One-year and 2-year survival rates were reported in 7 and 5 clinical studies involving 560 and 358 lung cancer patients, respectively (Figure 8). Fixed-effects models were applied because there was no significant heterogeneity for either 1-year or 2-year survival rate (I^2^ = 0%, *p* = 0.93; I^2^ = 0%, *p* = 0.67, Figure 8). The results demonstrated that the addition of elemene to chemotherapy significantly increased the 1-year and 2-year survival rates of lung cancer patients (RR:1.34, 95% CI:1.15–1.56, *p* = 0.0002; RR:1.57, 95% CI:1.14–2.16, *p* = 0.006, Figure 8). Sensitivity and meta-regression analysis confirmed that the pooled results would not be changed by any study or variable included in this article (Supplementary Figures S2S, T; *p* > 0.1; Supplementary Table S1). Based on TSA analysis, the cumulative Z curves reached the RIS or TSA boundaries, demonstrating the results were conclusive (Supplementary Figures S6P, Q).

### Quality of evidence

GRADEpro software was used to summarize the quality of evidence for the outcomes provided in Supplementary Table S2. The quality of evidence was moderate in 6 outcomes, low in 11, and very low in 8, which indicated that the inference of combination of elemene on response rate, DCR, leukopenia (Ⅲ-Ⅳ), hemoglobin reduction, neurotoxicity, and phlebitis was more credible.

## Discussion

The intricate nature of cancer cells continues to pose a significant challenge for researchers and medical professionals. Besides efficacy, the quality of life and psychological state of patients should also be fully considered during cancer treatment. Chemotherapy is a common treatment for cancer patients, but the side effects and multi-drug resistance problems that come with it cannot be ignored. Certain studies have indicated that chemotherapeutic drugs can induce alterations in the pulmonary microenvironment, thereby promoting the metastasis of cancer cells (Keklikoglou et al., 2019; Middleton et al., 2021). Therefore, adjuvant therapy is often used to achieve improved therapeutic outcomes and mitigate the problems caused by chemotherapy.

Elemene injection and elemene oral emulsion are applied in clinical in China for more than 20 years, the principal component, β-elemene has attracted researchers’ attention, and the molecular mechanisms for anticancer, reversing chemotherapeutic resistance, and alleviating neuropathic pain are revealed, involving Cyclin-dependent kinases, glycolytic kinases, ATP-binding cassette transporters, N6-methyladenosine methyltransferase, NMYC downstream-regulated gene 2, etc., (Zhao et al., 2011; Zhang et al., 2013b; Zhai et al., 2019; Liu et al., 2020; Ma et al., 2021). Most of all, there were no reported severe adverse effects so far. However, the appearance of a few dissenting voices has caught our attention. Whether the combination of elemene can enhance the efficacy and reduce the toxicity of different chemotherapy regimens for different cancers. For a variety of side effects caused by different chemotherapy regimens, whether the combination of elemene has a relief effect. In this study, a comprehensive literature search and reference selection were carried out to ensure that no relevant clinical studies were missed. GRADE system and TSA were used to assess the strength of evidence and robustness of our results. To ensure the accuracy of the results, we performed sensitivity, meta-regression, and subgroup analysis to find the source of heterogeneity and further analyzed the effect of the combination according to CNE, CT, CR, etc. Our study integrated 38 clinical studies encompassing a total of 2709 patients diagnosed with 12 different types of cancer and treated with 35 distinct chemotherapy regimens. The results of our study indicate that elemene could increase the efficacy, quality of life, immune function, and survival rate of patients undergoing chemotherapy, while also reducing the prevalence of most chemotherapy-induced side effects. However, significant improvements in response rate and DCR existed only when the cycle number of elemene was less than 6. Although regression analyses showed that the effects of elemene on most side effects, immune function, quality of life, and survival rate were not significantly influenced by SZ, CR, CT, TT, DE, CNE, and DDE, subgroup analysis indicated that CNE and TT were the primary contributors to heterogeneity in these findings. The effect of elemene on anemia and CD8^+^ T is influenced by CT, while the quality of life improvement and stability rate is affected by CR. Nevertheless, both GRADE and TSA suggested us more high-quality studies are needed to included obtain more precise conclusions. Unexpectedly, we found that prolonged administration of elemene leads to enhanced immune function, albeit with a potential decline in the improvement of the incidence of side effects of chemotherapy. However, due to the low quality of most of the outcomes on immune function and adverse effects, this conclusion needs to be supported by additional clinical data and deserves further attention. Notably, concomitant use of elemene in chemotherapy-treated cancer patients increased the incidence of phlebitis, but the result may alter with subsequent, more adequate clinical data. The quality of life of cancer patients was increased when elemene combined with cisplatin and docetaxel/vinorelbine, FOLFOX4, CTV, or paclitaxel and carboplatin, or no more than 6 cycles. In general, the treatment time and the number of cycles of elemene should be strictly controlled.

Regretfully, this study presents both strengths and limitations. Although all included studies were clinical trials, the quality of them was not high, with a majority lacking information on double-blind procedures. The existence of publication bias may also lead to bias in the evaluation of intervention effects. Our analysis discovered that the elemene combination therapy was regional, as elemene injection and elemene oral emulsion are independently developed and used in China. Consequently, the effect on chemotherapy patients in different countries or regions remains uncertain. What’s more, we found that the combination was mainly used in patients with gastric cancer and lung cancer, and the sample sizes of patients with breast cancer, liver cancer, acute myelocytic leukemia, colorectal cancer, and Non-Hodgkin lymphoma were so small that some results merely indicated tendencies without reaching statistical significance. Moreover, changes in serum-related indicators in cancer patients treated with chemotherapy have rarely been reported in studies. In addition, there are few studies about drug-resistant patients. Due to the limited number of studies included for some of the outcomes, it is difficult to ensure the accuracy of the conclusions. Therefore, the recommended plan in this study may not be optimal, however, it will clear up the confusion about the clinical use of this drug and provide a reference for the treatment of some cancer patients.

It is worth noting that while elemene injection and elemene oral emulsion share the same ingredient, the drug description indicates a notable reduction in the applicability of the oral emulsion, rendering it more suitable for the adjuvant treatment of esophageal cancer and gastric cancer (Bai et al., 2021). However, the difference in efficacy between elemene injection and elemene oral emulsion is still unknown. In our study, there were only two clinical studies that used elemene oral emulsion. The comparisons could not be made due to the lack of identical combination groups. Meanwhile, the absence of published clinical studies and systematic reviews on the comparative efficacy of elemene injection *versus* elemene oral emulsion for cancer treatment suggests that this is a good point for an in-depth study. The poor aqueous solubility and bioavailability of elemene limit its clinical application. Researchers focused on the secondary development of its major active ingredient, β-elemene to solve the problems of poor aqueous solubility, low bioavailability, and severe phlebitis, as well as to improve antitumor efficacy (Chen et al., 2017; Zhai et al., 2018). Although the structure modification and development of the delivery system have improved the antitumor activity and bioavailability of β-elemene to some extent, it is still in the biological experimental stage, and no new products have entered the clinic. Therefore, adjusting the treatment regimen may remain the main solution for now.

## Conclusion

Combination with elemene could increase the efficacy, quality of life, immune function, and survival rate of chemotherapy patients, and reduce the prevalence of most chemotherapy-induced side effects. A shorter duration and fewer cycles are recommended for its combination.

## Data Availability

Publicly available datasets were analyzed in this study. This data can be found here: PubMed, Cochrane Library, Web of Science, EMBASE, CKNI, Wan Fang, and VIP database, which can be obtained by searching according to the names and accession numbers.
