# Supplementary material for: Clinical benefit and risk of elemene in cancer patients undergoing chemotherapy: a systematic review and meta-analysis
Source: Front Pharmacol. 2023 Aug 2;14:1185987. doi: 10.3389/fphar.2023.1185987 (PMC10436211; doi:10.3389/fphar.2023.1185987)
Supplement: Supplementary file 1 [file Table2.docx]

**Table S2 GRADE profile for the quality of evidence**

| **Quality assessment** | | | | | | | **Summary of findings** | | | | | **Importance** |
| --- | --- | --- | --- | --- | --- | --- | --- | --- | --- | --- | --- | --- |
|  |  |  |  |  |  |  | **No of patients** | | **Effect** | | **Quality** |  |
| **No of studies** | **Design** | **Limitations** | **Inconsistency** | **Indirectness** | **Imprecision** | **Other considerations** | **Elemene combined with chemotherapy** | **chemotherapy** | **Relative (95% CI)** | **Absolute** |  |  |
| **Response rate** | | | | | | | | | | | | |
| 35 | randomised trials | serious^1^ | no serious | no serious | no serious | none | 772/1278 (60.4%) | 502/1229 (40.8%) | RR 1.48 (1.38 to 1.6) | 196 more per 1000 (from 155 more to 245 more) | ⊕⊕⊕O MODERATE | CRITICAL |
|  |  |  |  |  |  |  |  | 40% |  | 192 more per 1000 (from 152 more to 240 more) |  |  |
| **DCR** | | | | | | | | | | | | |
| 30 | randomised trials | serious^1^ | no serious | no serious | no serious | none | 915/1082 (84.6%) | 730/1036 (70.5%) | RR 1.2 (1.15 to 1.26) | 141 more per 1000 (from 106 more to 183 more) | ⊕⊕⊕O MODERATE | CRITICAL |
|  |  |  |  |  |  |  |  | 72.4% |  | 145 more per 1000 (from 109 more to 188 more) |  |  |
| **Leukopenia (Ⅲ-Ⅳ)** | | | | | | | | | | | | |
| 14 | randomised trials | serious^1^ | no serious | no serious | no serious | reporting bias^2^ strong association^3^ | 59/522 (11.3%) | 124/489 (25.4%) | RR 0.46 (0.35 to 0.61) | 137 fewer per 1000 (from 99 fewer to 165 fewer) | ⊕⊕⊕O MODERATE | IMPORTANT |
|  |  |  |  |  |  |  |  | 25% |  | 135 fewer per 1000 (from 98 fewer to 162 fewer) |  |  |
| **Thrombocytopenia** | | | | | | | | | | | | |
| 12 | randomised trials | serious^1^ | no serious | no serious | no serious | reporting bias^2^ | 233/468 (49.8%) | 262/445 (58.9%) | RR 0.86 (0.78 to 0.95) | 82 fewer per 1000 (from 29 fewer to 130 fewer) | ⊕⊕OO LOW | IMPORTANT |
|  |  |  |  |  |  |  |  | 65.6% |  | 92 fewer per 1000 (from 33 fewer to 144 fewer) |  |  |
| **Liver function damage** | | | | | | | | | | | | |
| 12 | randomised trials | serious^1^ | no serious ^1^ | no serious | no serious | reporting bias^2^ | 93/442 (21%) | 107/426 (25.1%) | RR 0.82 (0.68 to 1) | 45 fewer per 1000 (from 80 fewer to 0 more) | ⊕⊕OO LOW | IMPORTANT |
|  |  |  |  |  |  |  |  | 16.2% |  | 29 fewer per 1000 (from 52 fewer to 0 more) |  |  |
| **Digestive tract reactions** | | | | | | | | | | | | |
| 18 | randomised trials | serious^1^ | serious^4^ | no serious | no serious | reporting bias^2^ | 229/596 (38.4%) | 272/589 (46.2%) | RR 0.84 (0.72 to 0.97) | 74 fewer per 1000 (from 14 fewer to 129 fewer) | ⊕OOO VERY LOW | IMPORTANT |
|  |  |  |  |  |  |  |  | 52.8% |  | 84 fewer per 1000 (from 16 fewer to 148 fewer) |  |  |
| **Hemoglobin reduction** | | | | | | | | | | | | |
| 7 | randomised trials | serious^1^ | no serious | no serious | no serious | none | 151/298 (50.7%) | 173/278 (62.2%) | RR 0.83 (0.73 to 0.95) | 106 fewer per 1000 (from 31 fewer to 168 fewer) | ⊕⊕⊕O MODERATE | IMPORTANT |
|  |  |  |  |  |  |  |  | 62% |  | 105 fewer per 1000 (from 31 fewer to 167 fewer) |  |  |
| **Myelosuppression** | | | | | | | | | | | | |
| 8 | randomised trials | serious^1^ | very serious^5^ | no serious | no serious | none | 195/329 (59.3%) | 226/323 (70%) | RR 0.75 (0.53 to 1.05) | 175 fewer per 1000 (from 329 fewer to 35 more) | ⊕OOO VERY LOW | IMPORTANT |
|  |  |  |  |  |  |  |  | 66.5% |  | 166 fewer per 1000 (from 313 fewer to 33 more) |  |  |
| **Kidney function damage** | | | | | | | | | | | | |
| 4 | randomised trials | serious^1^ | very serious^5^ | no serious | no serious | reporting bias^2^ | 47/133 (35.3%) | 55/121 (45.5%) | RR 0.59 (0.26 to 1.37) | 186 fewer per 1000 (from 336 fewer to 168 more) | ⊕OOO VERY LOW | NOT IMPORTANT |
|  |  |  |  |  |  |  |  | 31.1% |  | 128 fewer per 1000 (from 230 fewer to 115 more) |  |  |
| **neurotoxicity** | | | | | | | | | | | | |
| 7 | randomised trials | serious^1^ | no serious | no serious | no serious | none | 54/277 (19.5%) | 69/285 (24.2%) | RR 0.81 (0.61 to 1.07) | 46 fewer per 1000 (from 94 fewer to 17 more) | ⊕⊕⊕O MODERATE | IMPORTANT |
|  |  |  |  |  |  |  |  | 25% |  | 48 fewer per 1000 (from 98 fewer to 18 more) |  |  |
| **phlebitis** | | | | | | | | | | | | |
| 4 | randomised trials | serious^1^ | no serious | no serious | no serious | reporting bias^2^ strong association^3^ | 20/157 (12.7%) | 6/165 (3.6%) | RR 3.41 (1.47 to 7.93) | 88 more per 1000 (from 17 more to 252 more) | ⊕⊕⊕O MODERATE | IMPORTANT |
|  |  |  |  |  |  |  |  | 3.9% |  | 94 more per 1000 (from 18 more to 270 more) |  |  |
| **anemia** | | | | | | | | | | | | |
| 5 | randomised trials | serious^1^ | no serious | no serious | no serious | reporting bias^2^ | 81/216 (37.5%) | 94/213 (44.1%) | RR 0.84 (0.7 to 1) | 71 fewer per 1000 (from 132 fewer to 0 more) | ⊕⊕OO LOW | NOT IMPORTANT |
|  |  |  |  |  |  |  |  | 41.7% |  | 67 fewer per 1000 (from 125 fewer to 0 more) |  |  |
| **CD4+ T cells (Better indicated by lower values)** | | | | | | | | | | | | |
| 9 | randomised trials | serious^1^ | very serious^5^ | no serious | no serious | reporting bias^2^ | 308 | 284 | - | MD 6.62 higher (4.99 to 8.24 higher) | ⊕OOO VERY LOW | IMPORTANT |
| **CD3+ T cells (Better indicated by lower values)** | | | | | | | | | | | | |
| 6 | randomised trials | serious^1^ | very serious^5^ | no serious | no serious | reporting bias^2^ | 213 | 204 | - | MD 6.48 higher (4.4 to 8.57 higher) | ⊕OOO VERY LOW | IMPORTANT |
| **CD8+ T cells (Better indicated by lower values)** | | | | | | | | | | | | |
| 8 | randomised trials | serious^1^ | very serious^5^ | no serious | no serious | none | 295 | 272 | - | MD 0.49 lower (2.59 lower to 1.6 higher) | ⊕OOO VERY LOW | IMPORTANT |
| **CD4+/CD8+ (Better indicated by lower values)** | | | | | | | | | | | | |
| 8 | randomised trials | serious^1^ | serious^6^ | no serious | no serious | none | 252 | 235 | - | MD 0.33 higher (0.24 to 0.42 higher) | ⊕⊕OO LOW | IMPORTANT |
| **The rate of quality-of-life improvement and stability** | | | | | | | | | | | | |
| 10 | randomised trials | serious^1^ | serious^6^ | no serious | no serious | none | 252/319 (79%) | 192/326 (58.9%) | RR 1.31 (1.12 to 1.53) | 183 more per 1000 (from 71 more to 312 more) | ⊕⊕OO LOW | IMPORTANT |
|  |  |  |  |  |  |  |  | 64.6% |  | 200 more per 1000 (from 78 more to 342 more) |  |  |
| **KPS (Better indicated by lower values)** | | | | | | | | | | | | |
| 4 | randomised trials | serious^1^ | very serious^5^ | no serious | no serious | reporting bias^2^ | 111 | 111 | - | MD 8.04 higher (3.87 to 12.21 higher) | ⊕OOO VERY LOW | IMPORTANT |
| **LCSS - anorexia score (Better indicated by lower values)** | | | | | | | | | | | | |
| 2 | randomised trials | serious^1^ | no serious | no serious | no serious | reporting bias^2^ | 86 | 86 | - | MD 3.37 lower (4.96 to 1.78 lower) | ⊕⊕OO LOW | NOT IMPORTANT |
| **LCSS - dyspnea score (Better indicated by lower values)** | | | | | | | | | | | | |
| 2 | randomised trials | serious^1^ | no serious | no serious | no serious | reporting bias^2^ | 86 | 86 | - | MD 3.62 lower (5.36 to 1.89 lower) | ⊕⊕OO LOW | NOT IMPORTANT |
| **LCSS - cough score (Better indicated by lower values)** | | | | | | | | | | | | |
| 2 | randomised trials | serious^1^ | no serious | no serious | no serious | reporting bias^2^ | 86 | 86 | - | MD 3.44 lower (4.58 to 2.31 lower) | ⊕⊕OO LOW |  |
| **LCSS - hemoptysis score (Better indicated by lower values)** | | | | | | | | | | | | |
| 2 | randomised trials | serious^1^ | no serious | no serious | no serious | reporting bias^2^ | 86 | 86 | - | MD 3.61 lower (5.97 to 1.26 lower) | ⊕⊕OO LOW |  |
| **LCSS - pain score (Better indicated by lower values)** | | | | | | | | | | | | |
| 2 | randomised trials | serious^1^ | no serious | no serious | no serious | reporting bias^2^ | 86 | 86 | - | MD 4 lower (5.53 to 2.48 lower) | ⊕⊕OO LOW |  |
| **1-year survival rate** | | | | | | | | | | | | |
| 7 | randomised trials | serious^1^ | no serious | no serious | no serious | reporting bias^2^ | 172/285 (60.4%) | 124/275 (45.1%) | RR 1.34 (1.15 to 1.56) | 153 more per 1000 (from 68 more to 253 more) | ⊕⊕OO LOW | IMPORTANT |
|  |  |  |  |  |  |  |  | 45% |  | 153 more per 1000 (from 67 more to 252 more) |  |  |
| **2-year survival rate** | | | | | | | | | | | | |
| 5 | randomised trials | serious^1^ | no serious | no serious | serious | reporting bias^2^ | 69/184 (37.5%) | 42/174 (24.1%) | RR 1.57 (1.14 to 2.16) | 138 more per 1000 (from 34 more to 280 more) | ⊕OOO VERY LOW | IMPORTANT |
|  |  |  |  |  |  |  |  | 20% |  | 114 more per 1000 (from 28 more to 232 more) |  |  |

^1^ most studies did not mention the allocation concealment method and whether the process was double-blind
^2^ asymmetric funnel plot or less than 6 studies.
^3^ RR >2 or <0.5
^4^ diminishing treatment effect with time
^5^ 75%≤I^2^≤100%
^6^ 50%≤I^2^<75%
