# Supplementary material for: Clinical benefit and risk of elemene in cancer patients undergoing chemotherapy: a systematic review and meta-analysis
Source: Front Pharmacol. 2023 Aug 2;14:1185987. doi: 10.3389/fphar.2023.1185987 (PMC10436211; doi:10.3389/fphar.2023.1185987)
Supplement: Supplementary file 2 [file DataSheet1.docx]

**Supplemental information**


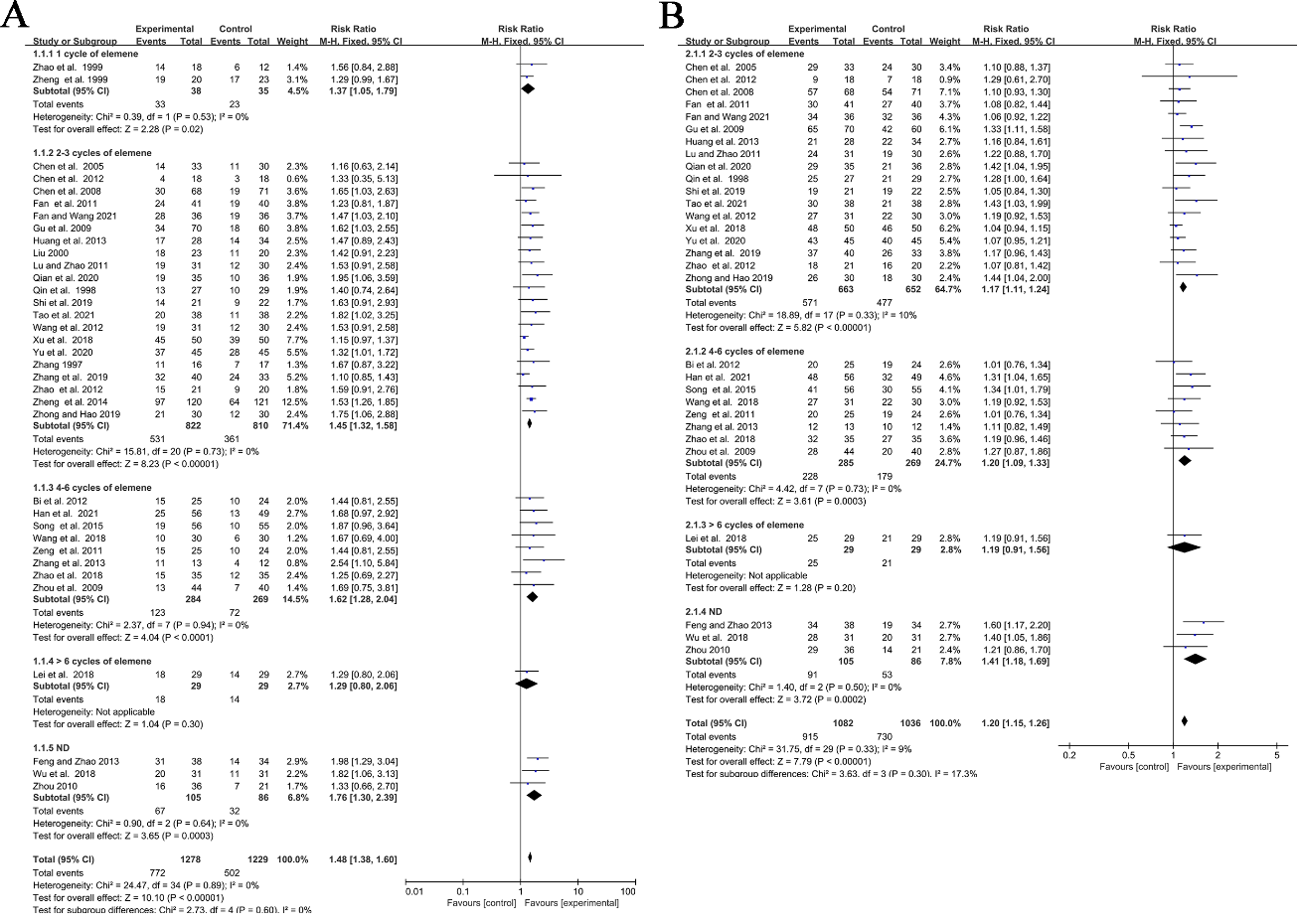


**Fig. S1 Forest plot describing the efficacy of elemene on the response rate (A) and DCR (B) of cancer patients treated with chemotherapy according to the cycle number of elemene.**


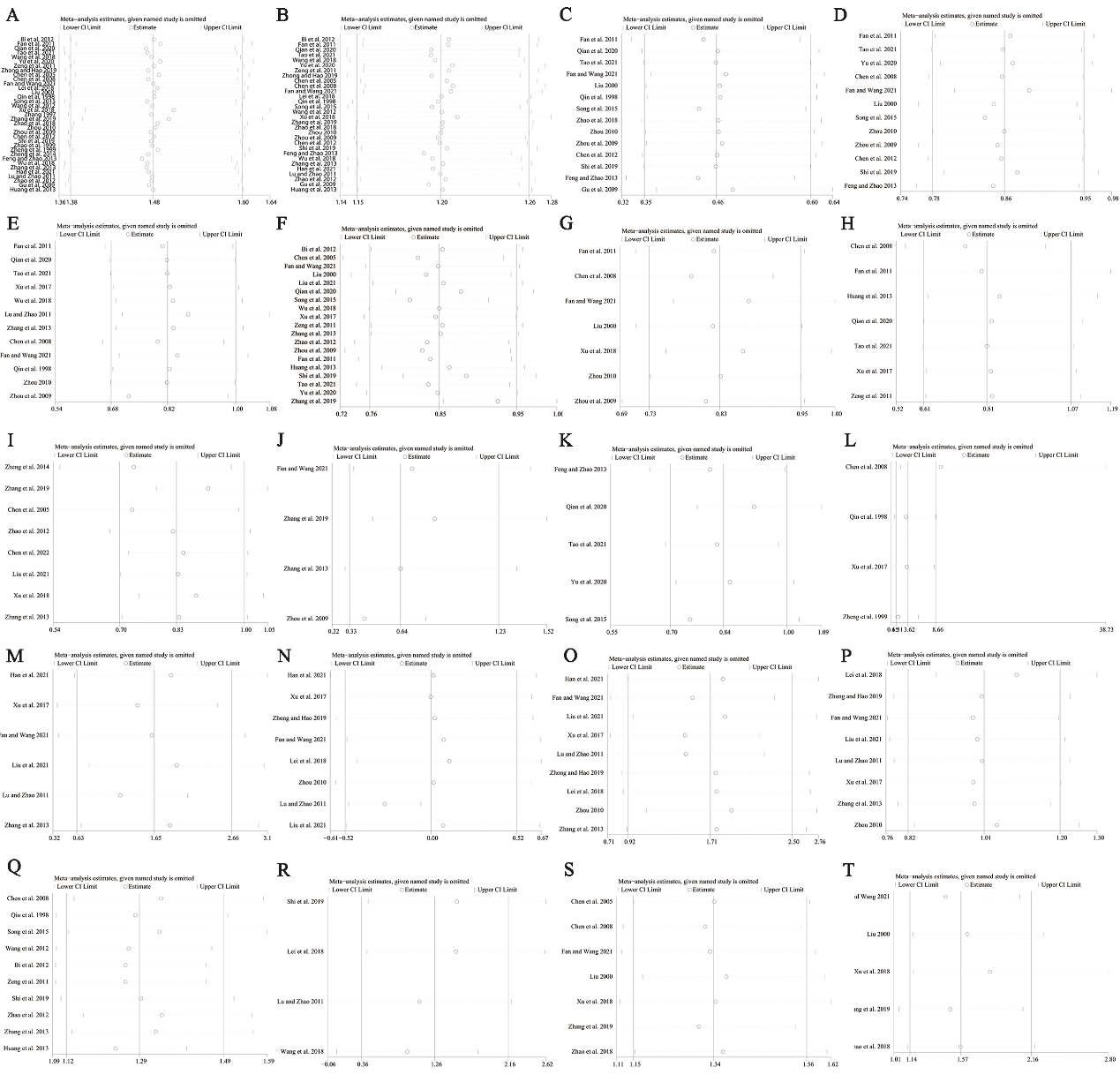


**Fig. S2 The plots of sensitivity analysis**

(A) Response rate, (B) DCR, (C) Leukophenia (Ⅲ-Ⅳ), (D) Thrombocytopenia, (E)Liver function damage, (F) Digestive tract reactions, (G) Hemoglobin reduction, (H) Neurotoxicity, (I) Myelosuppression, (J) Kidney function damage, (K) Anemia, (L) Phlebitis, (M) CD3^+^ T cells, (N), CD8^+^ T cells (O) CD4^+^ T cells, (P) CD4^+^/CD8^+^ T cells, (Q) Quality of life improvement and stability rate, (R) KPS, (S) one-year survival rate, (T) two-year survival rate


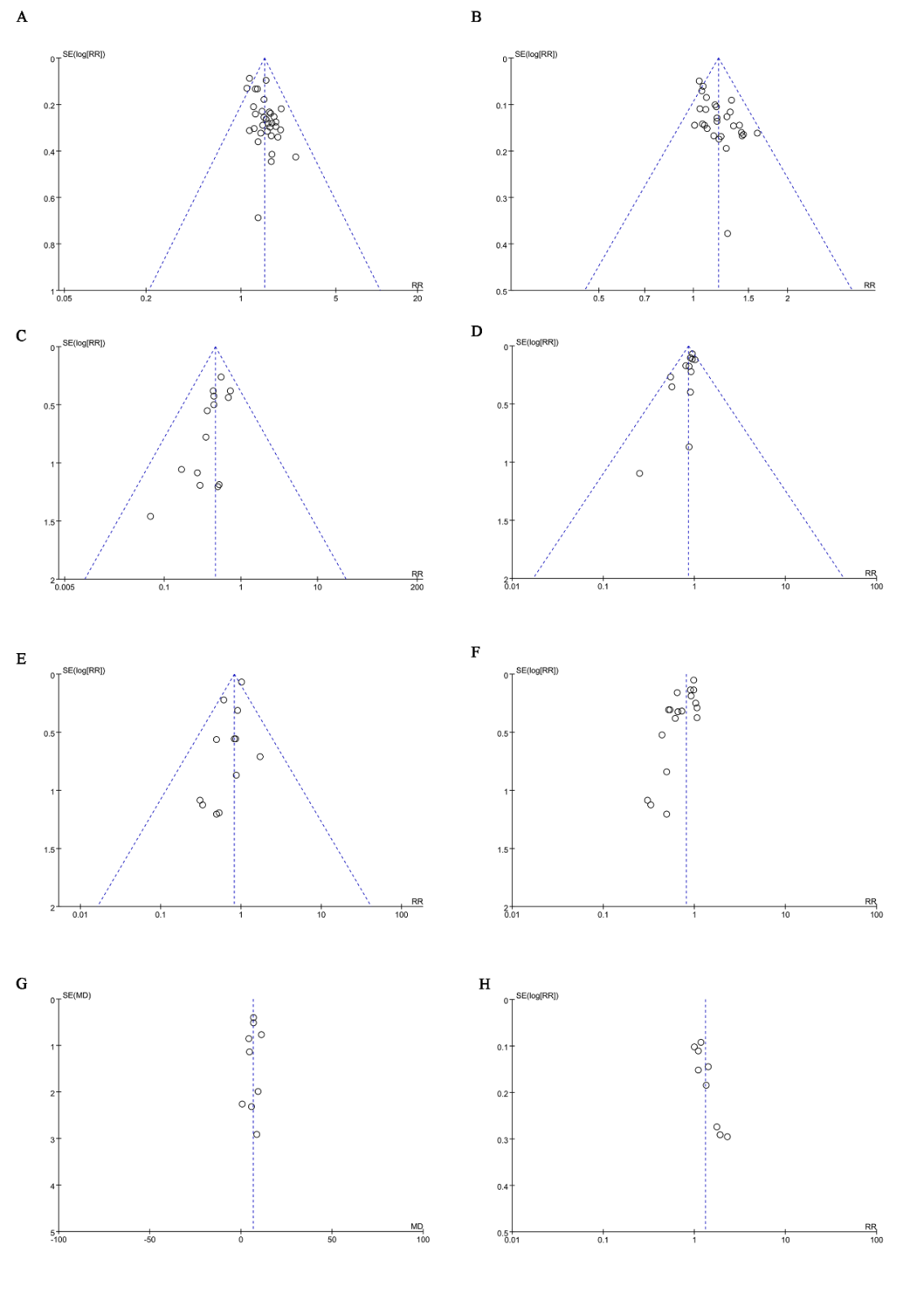


**Fig. S3 Funnel plot assessment of publication bias for the 7 outcomes**

1. Response rate, (B) DCR, (C) Leukophenia (Ⅲ-Ⅳ), (D) Thrombocytopenia, (E) Liver function damage, (F) Digestive tract reactions, (G) CD4^+^ T cells, (H) Quality of life improvement and stability rate


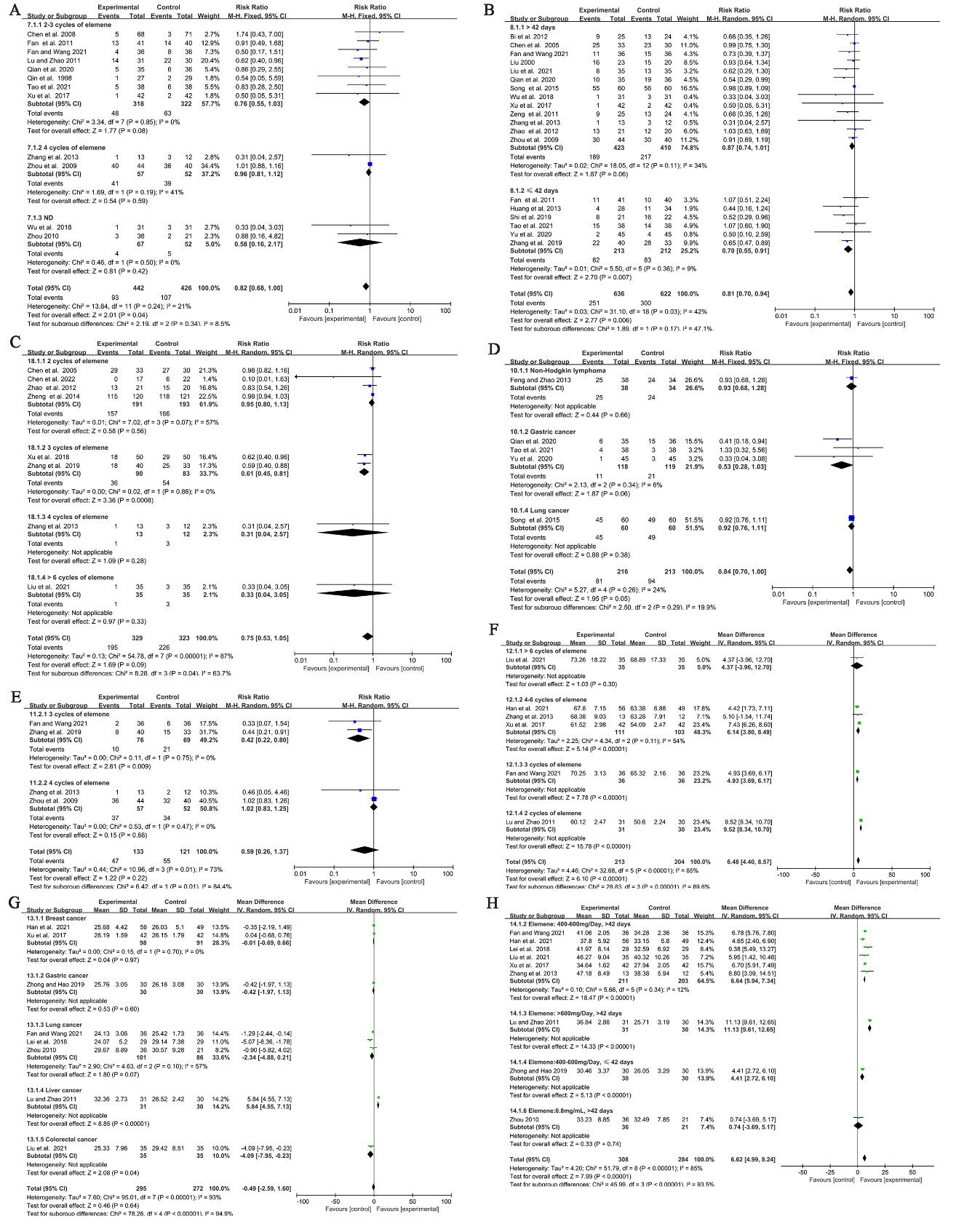

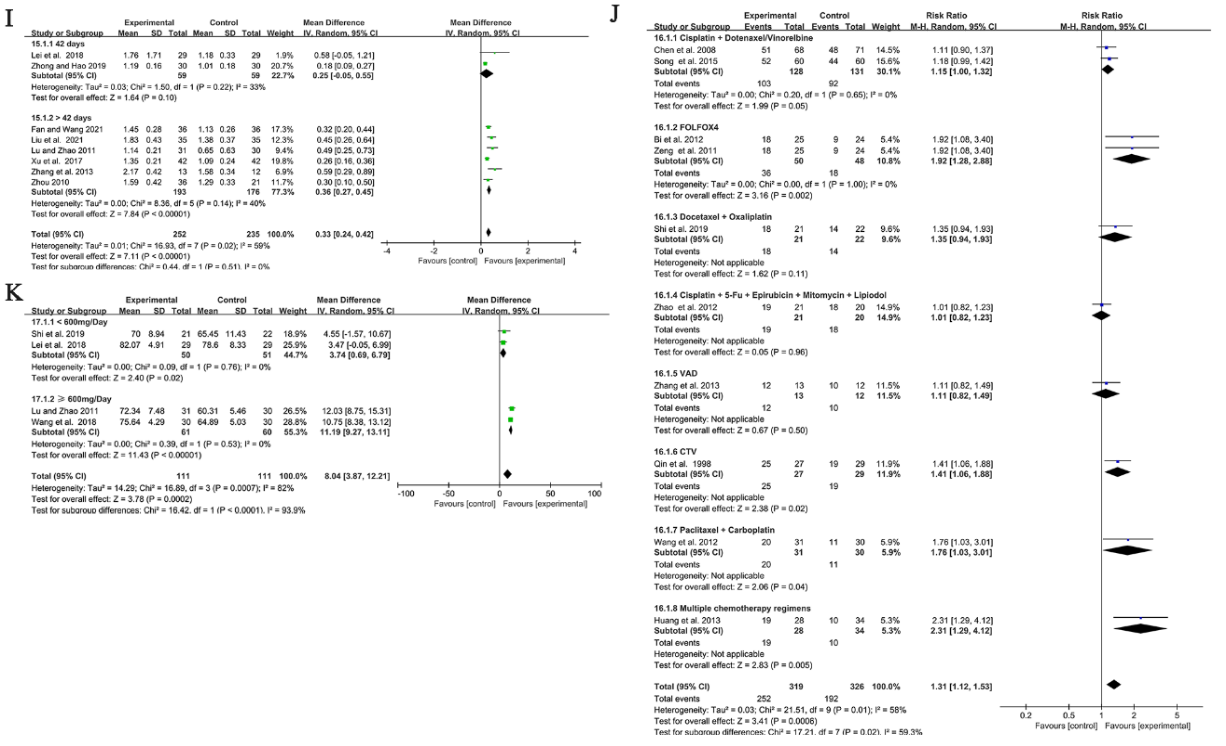


**Fig. S4 The plots of subgroup analysis**

(A) Liver function damage, (B) Digestive tract reactions, (C) Myelosuppression, (D) Anemia, (E) Kidney function damage, (F) CD3+ T cells, (G) CD8+ T cells, (H) CD4+ T cells, and (I) CD4+/CD8+ T cells, (J) Quality of life improvement and stability rate, (K) KPS


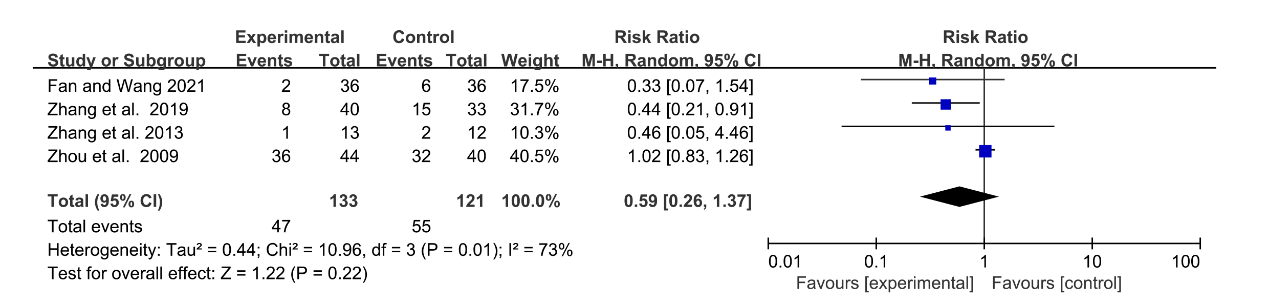


**Fig. S5 The influence of elemene on the Kidney function impairment of chemotherapy patients**


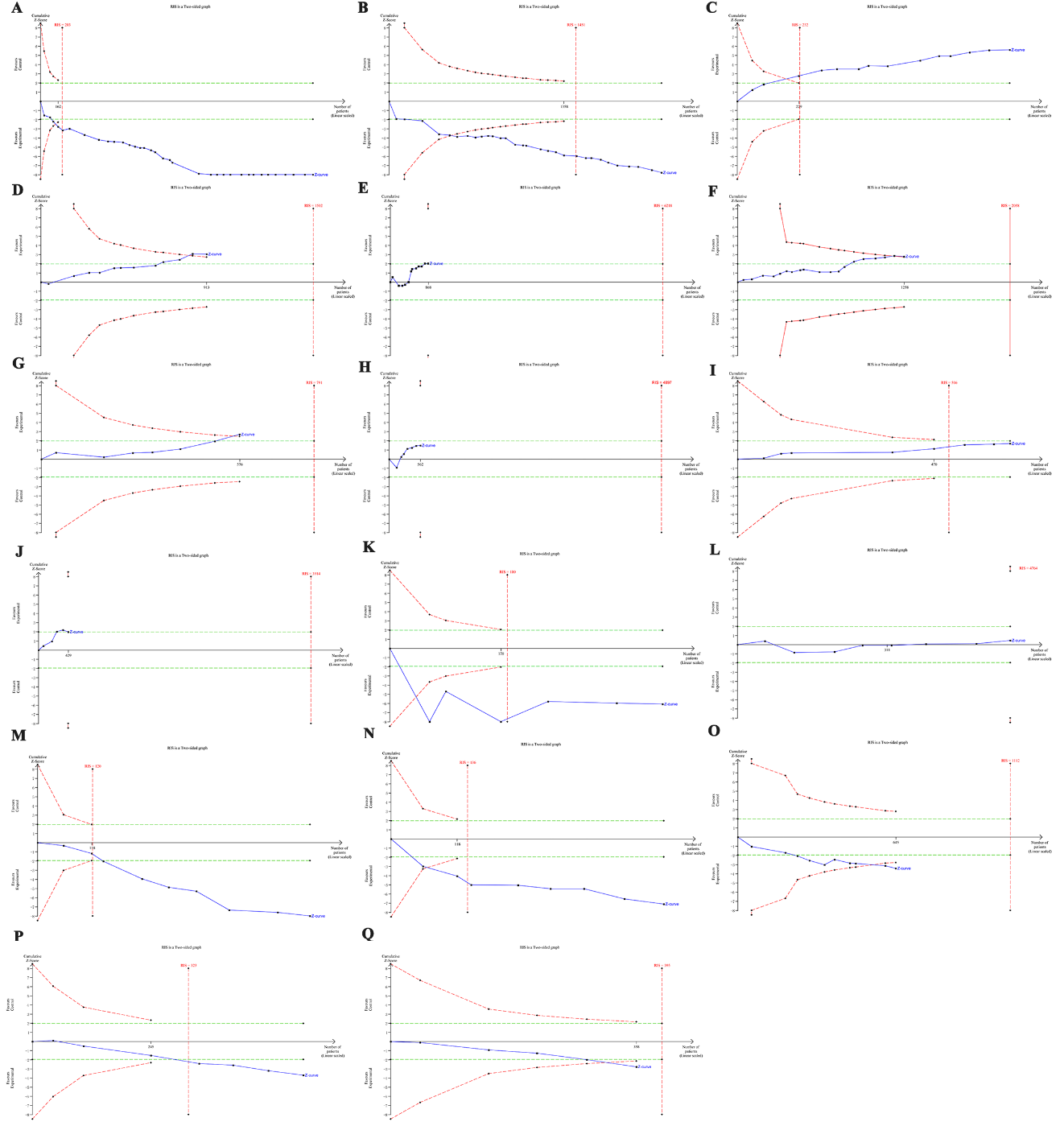


**Fig. S6 TSA results of elemene combined with chemotherapy versus chemotherapy alone**

(A) Response rate, (B) DCR, (C) Leukopenia (Ⅲ-Ⅳ), (D) Thrombocytopenia, (E) Liver function damage, (F) Digestive tract reactions, (G) Hemoglobin reduction, (H) Neurotoxicity, (I) myelosuppression, (J) Anemia, (K) CD3+ T cells, (L) CD8^+^ T cells (M) CD4^+^ T cells, (N) CD4^+^/CD8^+^, (O) The rate of quality-of-life improvement and stability, (P) One-year survival rate, (Q) Two-year survival rate

**Table S1 The results of the Meta-regression analysis**

| **Outcome** | | **Variables** | **Coefficient** | **95% CI lower** | **95% CI upper** | **P** |
| --- | --- | --- | --- | --- | --- | --- |
| **Response rate** | | SZ | 0.027 | -0.117 | 0.170 | 0.708 |
|  |  | CR | -0.003 | -0.017 | 0.012 | 0.703 |
|  |  | TT | 0.049 | -0.137 | 0.236 | 0.591 |
|  |  | CT | -0.004 | -0.056 | 0.048 | 0.872 |
|  |  | DE | 0.006 | -0.029 | 0.042 | 0.723 |
|  |  | CNE | 0.102 | -0.014 | 0.218 | 0.082 |
|  |  | DDE | 0.174 | -0.295 | 0.642 | 0.453 |
| **DCR** | | SZ | -0.096 | -0.192 | 0.000 | 0.050 |
|  |  | CR | -0.001 | -0.010 | 0.007 | 0.719 |
|  |  | TT | 0.017 | -0.089 | 0.124 | 0.736 |
|  |  | CT | 0.004 | -0.026 | 0.035 | 0.765 |
|  |  | DE | -0.003 | -0.023 | 0.017 | 0.762 |
|  |  | CNE | 0.078 | 0.014 | 0.141 | 0.019 |
|  |  | DDE | 0.108 | -0.127 | 0.343 | 0.350 |
| **Side effects** | Leukopenia (Ⅲ-Ⅳ) | SZ | -0.099 | -1.501 | 1.303 | 0.869 |
|  |  | CR | -0.043 | -0.359 | 0.274 | 0.753 |
|  |  | TT | 0.174 | -1.233 | 1.582 | 0.772 |
|  |  | CT | -0.396 | -1.466 | 0.673 | 0.400 |
|  |  | DE | -0.066 | -0.318 | 0.185 | 0.542 |
|  |  | CNE | 0.683 | -0.494 | 1.860 | 0.206 |
|  |  | DDE | 0.034 | -2.667 | 2.735 | 0.976 |
|  | Thrombocytopenia | SZ | -0.267 | -1.324 | 0.789 | 0.521 |
|  |  | CR | -0.066 | -0.278 | 0.145 | 0.434 |
|  |  | TT | -0.628 | -2.655 | 1.398 | 0.438 |
|  |  | CT | -0.175 | -0.941 | 0.590 | 0.560 |
|  |  | DE | -0.034 | -0.264 | 0.196 | 0.704 |
|  |  | CNE | -0.065 | -0.732 | 0.603 | 0.801 |
|  |  | DDE | 0.095 | -1.415 | 1.605 | 0.870 |
|  | Damage to liver function | SZ | -0.773 | -3.985 | 2.439 | 0.541 |
|  |  | CR | 0.021 | -0.353 | 0.395 | 0.882 |
|  |  | TT | 0.249 | -2.608 | 3.105 | 0.821 |
|  |  | CT | -0.140 | -0.831 | 0.552 | 0.604 |
|  |  | DE | 0.026 | -0.653 | 0.705 | 0.920 |
|  |  | CNE | 0.372 | -0.764 | 1.508 | 0.415 |
|  |  | DDE | -0.228 | -3.888 | 3.432 | 0.871 |
| **Outcome** | | **Variables** | **Coefficient** | **95% CI lower** | **95% CI upper** | **P** |
| **Side effects** | Digestive tract reactions | SZ | -0.164 | -0.401 | 0.073 | 0.157 |
|  |  | CR | -0.013 | -0.065 | 0.039 | 0.593 |
|  |  | TT | -0.422 | -0.828 | -0.016 | 0.043 |
|  |  | CT | 0.042 | -0.105 | 0.188 | 0.543 |
|  |  | DE | -0.023 | -0.098 | 0.052 | 0.513 |
|  |  | CNE | -0.095 | -0.460 | 0.269 | 0.577 |
|  |  | DDE | 0.415 | -0.356 | 1.187 | 0.261 |
|  | Hemoglobin reduction | SZ | 0.355 | -2.296 | 3.006 | 0.339 |
|  |  | CR | -0.052 | -0.939 | 0.834 | 0.590 |
|  |  | TT | -0.193 | -6.590 | 6.204 | 0.767 |
|  |  | CT | -1.004 | -10.340 | 8.332 | 0.402 |
|  |  | DE | 0.448 | -2.219 | 3.116 | 0.279 |
|  | Neurotoxicity | SZ | -0.476 | -4.001 | 3.048 | 0.620 |
|  |  | CR | -0.019 | -1.467 | 1.428 | 0.959 |
|  |  | TT | 0.041 | -1.872 | 1.954 | 0.935 |
|  |  | CT | -0.098 | -1.230 | 1.035 | 0.746 |
|  |  | DE | 0.117 | -0.234 | 0.469 | 0.366 |
|  |  | CNE | -0.533 | -2.203 | 1.136 | 0.384 |
|  |  | DDE | -0.428 | -2.800 | 1.945 | 0.607 |
|  | Anemia | SZ | -1.520 | -5.870 | 2.830 | 0.272 |
|  |  | CR | -0.512 | -2.061 | 1.038 | 0.291 |
|  |  | TT | 0.447 | -2.773 | 3.667 | 0.611 |
|  |  | CT | 0.313 | -0.851 | 1.477 | 0.367 |
|  |  | DE | 0.742 | -5.446 | 6.929 | 0.370 |
|  |  | CNE | 0.789 | -4.691 | 6.269 | 0.318 |
|  |  | DDE | -0.307 | -12.681 | 12.067 | 0.805 |
|  | Kidney function damage | SZ | -0.300 | -24.222 | 23.623 | 0.899 |
|  |  | CR | -0.018 | -8.329 | 8.293 | 0.982 |
|  |  | TT | -0.550 | -11.152 | 10.052 | 0.629 |
|  |  | CT | -0.503 | -18.015 | 17.010 | 0.777 |
|  |  | DE | 0.796 | -13.973 | 15.565 | 0.618 |
|  |  | CNE | 0.098 | -15.208 | 15.405 | 0.948 |
|  | Phlebitis | SZ | 2.490 | -32.726 | 37.707 | 0.534 |
|  |  | CR | -0.719 | -23.902 | 22.464 | 0.761 |
|  |  | TT | -1.255 | -15.204 | 12.695 | 0.458 |
|  |  | CT | -1.206 | -8.740 | 6.328 | 0.291 |
|  |  | DE | -1.001 | -7.531 | 5.530 | 0.302 |
|  |  | CNE | 0.254 | -14.304 | 14.812 | 0.861 |
| **Outcome** | | **Variables** | **Coefficient** | **95% CI lower** | **95% CI upper** | **P** |
| **Side effects** | Myelosuppression | SZ | -0.987 | -3.509 | 1.536 | 0.302 |
|  |  | CR | -0.464 | -1.403 | 0.475 | 0.214 |
|  |  | TT | 0.232 | -1.038 | 1.501 | 0.602 |
|  |  | CT | -0.684 | -2.142 | 0.773 | 0.232 |
|  |  | DE | 0.004 | -0.111 | 0.118 | 0.939 |
|  |  | CNE | -0.471 | -0.821 | -0.121 | 0.018 |
| **Immunocyte** | CD3^+^ T cells | SZ | -0.844 | -26.572 | 24.884 | 0.749 |
|  |  | CR | 0.351 | -5.231 | 5.933 | 0.571 |
|  |  | CT | 1.076 | -8.795 | 10.948 | 0.398 |
|  |  | DE | 1.918 | -10.898 | 14.733 | 0.308 |
|  |  | CNE | -1.359 | -3.272 | 0.553 | 0.120 |
|  | CD8^+^ T cells | SZ | 0.485 | -23.350 | 24.320 | 0.839 |
|  |  | CR | -0.313 | -3.320 | 2.694 | 0.412 |
|  |  | TT | -0.311 | -20.735 | 20.113 | 0.878 |
|  |  | CT | 0.192 | -5.768 | 6.151 | 0.753 |
|  |  | DE | 0.158 | -5.059 | 5.375 | 0.766 |
|  |  | CNE | -0.160 | -6.346 | 6.026 | 0.798 |
|  | CD4^+^ T cells | SZ | -0.876 | -8.553 | 6.802 | 0.672 |
|  |  | CR | -0.011 | -0.971 | 0.949 | 0.965 |
|  |  | TT | -1.344 | -9.320 | 6.633 | 0.544 |
|  |  | CT | 0.458 | -1.867 | 2.783 | 0.486 |
|  |  | DE | 0.093 | -1.844 | 2.030 | 0.855 |
|  |  | CNE | -1.025 | -3.441 | 1.392 | 0.210 |
|  | CD4+/CD8+ T cells | SZ | 0.377 | -7.062 | 7.817 | 0.635 |
|  |  | CR | -0.045 | -0.773 | 0.683 | 0.574 |
|  |  | TT | -0.190 | -4.858 | 4.478 | 0.696 |
|  |  | CT | -0.088 | -1.439 | 1.263 | 0.560 |
|  |  | DE | -0.249 | -2.161 | 1.664 | 0.347 |
|  |  | CNE | -0.253 | -1.807 | 1.301 | 0.287 |
| **1-year survival rate** | | SZ | -0.140 | -3.502 | 3.222 | 0.690 |
|  |  | CR | 0.022 | -0.849 | 0.893 | 0.800 |
|  |  | TT | 0.121 | -3.071 | 3.312 | 0.715 |
|  |  | DE | -0.031 | -2.483 | 2.420 | 0.897 |
|  |  | CNE | -0.085 | -3.859 | 3.689 | 0.823 |
| **Outcome** | | **Variables** | **Coefficient** | **95% CI lower** | **95% CI upper** | **P** |
| **2-year survival rate** | | SZ | -0.310 | -8.244 | 7.624 | 0.707 |
|  |  | CR | 0.166 | -1.574 | 1.906 | 0.439 |
|  |  | TT | 0.496 | -5.615 | 6.608 | 0.490 |
|  |  | DE | 0.051 | -1.686 | 1.788 | 0.931 |
|  |  | CNE | 0.051 | -1.686 | 1.788 | 0.931 |
| **Quality of life improvement and stability rate** | | SZ | 0.419 | -0.216 | 1.053 | 0.105 |
|  |  | CR | 0.132 | -0.046 | 0.310 | 0.085 |
|  |  | TT | 0.149 | -0.734 | 1.032 | 0.544 |
|  |  | CT | 0.139 | -0.229 | 0.508 | 0.245 |
|  |  | DE | 0.118 | -0.201 | 0.437 | 0.252 |
|  |  | CNE | -0.228 | -0.917 | 0.462 | 0.291 |
|  |  | DDE | -0.393 | -2.076 | 1.291 | 0.421 |
| **KPS** | | SZ | -1.329 | -17.749 | 15.091 | 0.491 |
|  |  | CR | -0.349 | -6.681 | 5.983 | 0.611 |
|  |  | TT | -2.943 | -11.301 | 5.416 | 0.140 |
|  |  | CT | -0.920 | -4.031 | 2.191 | 0.166 |
|  |  | DE | 0.900 | -3.471 | 5.270 | 0.232 |
|  |  | CNE | 0.629 | -5.235 | 6.493 | 0.403 |
|  |  | DDE | -1.090 | -5.750 | 3.570 | 0.420 |

SZ: Sample size, CR: Chemotherapy regimen, TT: Treatment time, CT: Cancer type, DE: Dosage of Elemene, CNE: Cycle number of Elemene, DDE: Drug delivery of Elemene
